# Factors influencing the utilization of Traditional Chinese Medicine in cancer treatment: a qualitative meta-synthesis of patient and healthcare professional perspectives

**DOI:** 10.3389/fmed.2025.1501918

**Published:** 2025-03-05

**Authors:** Runbing Xu, Yanan Sun, Yifei Liu, Jiajun Pan, Yingying Han, Xinyu Zhang, Hequn Zhao, Mengfei Li, Yu Wu, Changhe Yu, Miao Jiang

**Affiliations:** ^1^Department of Hematology and Oncology, Dongzhimen Hospital, Beijing University of Chinese Medicine, Beijing, China; ^2^Xuanwu Hospital, Capital Medical University, Beijing, China; ^3^Girton College, University of Cambridge, Cambridge, United Kingdom; ^4^Department of Pulmonary Medicine, FangShan Hospital, Beijing University of Chinese Medicine, Beijing, China; ^5^Department of Tuina and Pain Management, Dongzhimen Hospital, Beijing University of Chinese Medicine, Beijing, China; ^6^School of Life Sciences, Beijing University of Chinese Medicine, Beijing, China

**Keywords:** Traditional Chinese Medicine, meta-synthesis, integrated oncology, implementation barriers, modifiable determinants

## Abstract

**Background:**

The complementary role of Traditional Chinese Medicine (TCM) in cancer management has been widely acknowledged. However, its implementation continues to face numerous challenges. Identifying and elucidating the factors that influence the integration of TCM into cancer therapy is essential. Developing effective implementation strategies is crucial to transitioning from theoretical evidence to practical application.

**Methods:**

A total of nine databases were systematically searched from their inception until 1 October 2023. The review includes qualitative and mixed-method studies examining the attitudes and perceptions of patients and healthcare providers toward the use of TCM in cancer treatment. The studies included were evaluated using a quality assessment tool. An appropriate model or framework was to identify potential facilitators and impediments affecting TCM implementation. Based on the identified barriers, potential behavior change interventions were subsequently developed.

**Results:**

A total of 39 studies are included in the review, comprising 31 qualitative and eight mixed-methods studies. The quality of these studies is acceptable. Key barriers to the implementation of TCM were identified as follows: insufficient knowledge and experience in TCM, neglect of details in doctor-patient communication, limited number of specialists, lack of funding, and absence of a multidisciplinary collaborative atmosphere. In response to these barriers, we recommend improving structured referral pathways, developing a structured communication manual, and other targeted interventions to enhance the integration of TCM in cancer care.

**Conclusion:**

This study identifies 48 factors that influence the implementation of TCM and tentatively proposes a series of intervention strategies. Future research should focus on localized empirical studies of factors and strategies in different healthcare settings.

**Systematic review registration:**

https://www.crd.york.ac.uk/PROSPERO/display_record.php?RecordID=421822, identifier CRD42023421822.

## 1 Introduction

Traditional Chinese Medicine (TCM), including diverse modalities such as herbal medicines, dietary therapy, acupuncture, Qigong, and therapeutic massage, constitutes a critical component of Traditional, Complementary, and Integrative Medicine (TCI) (1, 2). Recently, the role of TCM in integrative oncology (IO) care has garnered increasing attention (3). Certain TCM therapies, such as acupuncture, acupressure, and Qigong, have been supported by a range of clinical evidence for their positive effects in cancer supportive care (4–6), particularly in alleviating cancer-related symptoms, reducing treatment side effects, and improving quality of life (7). Authoritative clinical guidelines also recommend these therapies for reducing the adverse effects of conventional treatments. For example, the ASCO-SIO guidelines strongly recommend low- to moderate-intensity Tai Chi and Qigong for cancer-related fatigue (8). In contrast, the ESO-ESMO guidelines endorse acupuncture for managing menopausal hot flashes, chemotherapy-induced nausea, vomiting, and fatigue (9). Moreover, an increasing body of research provides preliminary evidence for the role of herbal medicine in enhancing the efficacy of conventional cancer treatments. For instance, a study involving 1,052 patients with hepatocellular carcinoma found that those receiving adjunctive herbal therapy had significantly higher overall survival rates at 1, 3, and 5 years than compared to those receiving conventional treatment alone (10). Another randomized, double-blind, placebo-controlled trial involving 354 EGFR mutation-positive advanced lung adenocarcinoma patients showed that the combination of herbal medicine and EGFR-TKI treatment significantly improved median progression-free survival (PFS) compared to EGFR-TKI monotherapy (11).

Although the evidence above aforementioned evidence indicates the unique role of TCM in adjunctive cancer care, its actual clinical application remains insufficient. One study showed that only 33.3% of American cancer patients used TCI in the past year, with acupuncture at just 2.0%, compared to 64% TCM utilization among Chinese cancer patients (12, 13). This disparity reflects differing efficacy perceptions, cultural influences, and challenges in promoting TCM across patients, healthcare providers (HCPs), healthcare systems, and society. Some original studies have also identified barriers to the clinical practice of TCM in oncology, such as low awareness among oncologists (14), limited related discussions with patients (15), and inadequate insurance coverage (16). However, no comprehensive analysis has addressed the facilitators and barriers to implementing TCM in integrated cancer care.

To fill this gap, this study analyzes the literature on patient and HCP attitudes toward TCM in cancer care, summarizing key facilitators and barriers to its implementation. Recognizing that stakeholder perceptions influence intervention adoption (17), we propose general intervention strategies based on an implementation science framework to promote the application of TCM in supportive cancer care.

## 2 Methods

### 2.1 Design

This study integrates evidence from published qualitative studies through qualitative meta-synthesis; the PROSPERO registration number is CRD42023421822. This study did not involve ethical approval and was reported adhering to the PRISMA guideline (18) (Supplementary Table 1).

### 2.2 Data sources and literature search

Considering the geographical nature of TCM, we mainly searched English and Chinese databases, and we searched a total of nine databases, including five English databases: Cochrane, PubMed, Embase, Web of Science, CINAHL, and four Chinese data China National Knowledge Infrastructure (CNKI), Wanfang Medicine Online, Chinese Scientific Journal Database (VIP) and SinoMed. The search was conducted from the database construction to 1 October 2023. The search strategy is described in the Supplementary Table 2.

### 2.3 Inclusion/exclusion criteria

The phenomenon of interest was the attitudes of cancer patients, their dependent caregivers, and Health Professionals toward TCM for cancer treatment, broadly including views, opinions, and experiences. We developed the following detailed inclusion-exclusion criteria (Table 1).

**TABLE 1:** Inclusion and exclusion criteria.

	Inclusion	Exclusion
Participants	Diagnosed patients with any tumor (regardless of the type of tumor, stage of disease, etc.)	Mixed samples from tumor and non-tumor patients
	Family members or caregivers of tumor patients lacking verbal communication abilities and autonomous decision-making capacity (e.g., Pediatric Cancer Patients, patients with severe cognitive disabilities, etc.)	
	Health professionals with relevant experience in TCM oncology treatment	
Interventions	Herbs and various preparations, acupuncture, massage, diet, Tai Chi, and many other traditional Chinese medicine therapies	Research on complementary alternative therapies for which data on traditional Chinese medicine therapies cannot be extracted separately
Types of study	Qualitative research, Qualitative results section of mixed studies	Studies where qualitative methods collected data but results were not analyzed qualitatively
	Full-text publication in English or Chinese	Conference papers, review articles, editorials, letters
	Original research	Repeat publication

### 2.4 Study selection and data extraction

The two authors carried out the screening process independently (Xu and Sun), and disputes were resolved by a third party (Jiang). Literature screening was performed using Endnote X9, independently by two authors (Zhang and Pan), with initial screening by title and abstract, followed by reading the full text to determine the final inclusion of articles. The differences in the summary of results were finalized by a third party (Yu). Information was extracted from the included articles using Excel based on the study purpose, including (1) general characteristics, (2) characteristics of the study population, (3) research objectives, (4) research design and theory, (5) data collection methods and analysis methods, and (6) final themes and conclusions.

### 2.5 Quality assessment

Two authors (Sun and Liu) independently assessed the methodology of the articles included in this study through a 10-item Critical Appraisal Skills Program (CASP) (19), and dissent was agreed upon through discussion with (Han). The CASP tool consists of 10 terms (see Supplementary Table 3), with entry scores of 0-No, 0.5-Can’t Tell, and 1-Yes, which allows for a complete assessment of the rigor, relevance, and reliability of the study (20, 21). The quality assessment does not exclude any study but only aims to increase research transparency.

### 2.6 Data synthesis

To achieve the research goals, this study applied the “best-fit” framework synthesis method (22) and thematic synthesis (23) for data integration and analysis. The process, conducted by four researchers, followed a sequential combination of deductive and inductive approaches: (1) *A priori* framework selection: Based on similar studies identifying behavioral barriers and facilitators (24–26), the Theoretical Domains Framework (TDF) (27) and the Capability-Opportunity-Motivation-Behaviour (COM-B) (28) model were selected as the *a priori* frameworks for this study. TDF categorizes behavioral determinants into 14 domains, addressing the complexity of implementing TCM in oncology care, while COM-B simplifies this process by mapping determinants into three key components: Capability, Opportunity, and Motivation. These frameworks provide a comprehensive approach for analyzing the factors influencing the application of TCM in cancer care; (2) Line-by-line coding: Extracted barrier and facilitator data from the literature through line-by-line coding; (3) Code aggregation: Categorized and summarized codes to identify factors influencing TCM use in oncology care; (4) Mapping and classification: Mapped barriers and facilitators to COM-B components and TDF domains, with unmapped factors marked as potential new themes; (5) Theme extraction and framework refinement: Synthesized factors into theme labels using thematic analysis, extending the TDF framework to include unmatched theme. The differences were resolved through team consensus, and one author (Li) conducted the final code review.

## 3 Results

### 3.1 Study characteristics

**FIGURE 1:**
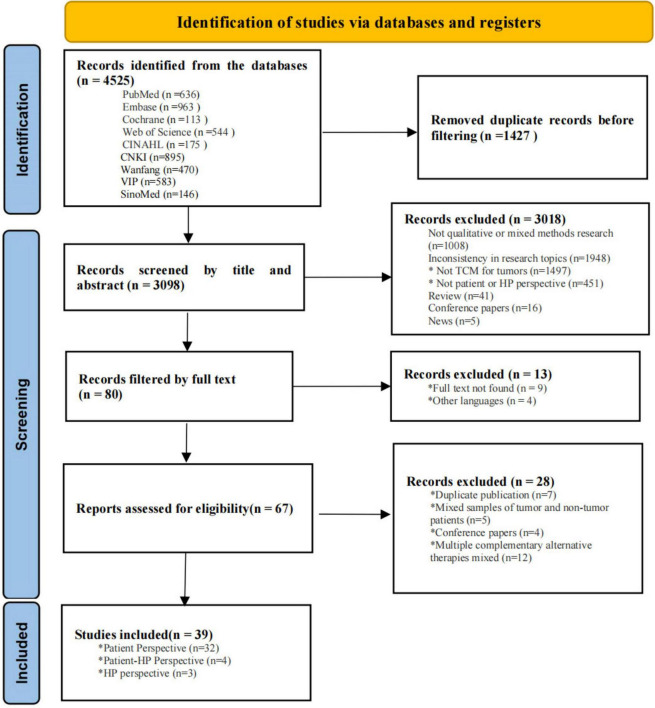
PRISMA flowchart of initial searches and inclusion.

**FIGURE 2:**
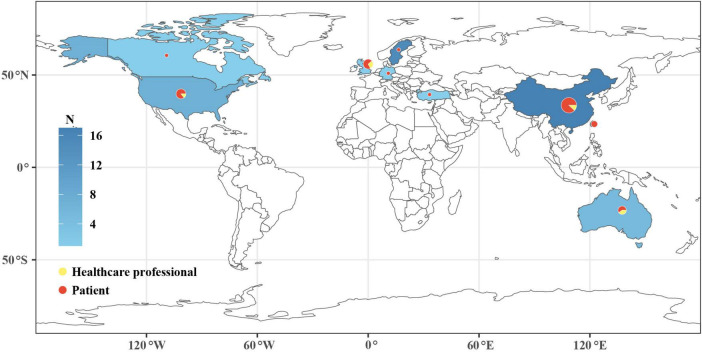
The geographical location of studies.

A total of 4,525 articles were retrieved, with 39 studies included: 31 qualitative and eight mixed-methods (see Figure 1 for the PRISMA flowchart). These studies were conducted in nine countries and regions, mostly China, United States, and United Kingdom (Figure 2). A total of 849 individuals were involved, including 813 patients and 36 health professionals. Key therapeutic modalities included acupuncture, herbal medicine, Qi Gong, TCM dietary therapy, and massage. Western countries focused on non-drug therapies, while East Asia emphasized comprehensive TCM approaches. Data collection relied on interviews and open-ended questionnaires, with analysis using content, thematic, and grounded theory methods. The characteristics of the included literature are shown in the Supplementary Table 4.

### 3.2 Methodological quality

The methodological evaluation using the CASP tool is shown in Table 2. The results indicated that majority of the studies (*n* = 28, 71.8%) met at least the 8/10 criteria, so the evidence was considered credible. Most studies (*n* = 33, 84.6%) provided the necessary elaboration on data analysis, while some studies (*n* = 24, 61.5%) missed reporting the researcher-participant relationship. In addition, few studies discussed how the researcher responded to problems during data collection.

**TABLE 2:** Quality assessment results.

References	1	2	3	4	5	6	7	8	9	10	Score
Billhult et al. (39)	Yes	Yes	Yes	Yes	Yes	Yes	Can’t tell	No	Yes	Can’t tell	8
Burrows Simpson (48)	Yes	Yes	Yes	Yes	Yes	Can’t tell	Yes	No	Yes	Yes	8.5
Chan et al. (35)	Yes	Yes	Yes	Yes	Yes	Can’t tell	Yes	Can’t tell	Yes	Yes	9
Chen et al. (42)	Yes	Yes	Yes	Yes	Yes	No	Yes	Can’t tell	Yes	Yes	8.5
De Valois et al. (33)	Yes	Yes	Yes	Can’t tell	Yes	Yes	Yes	Can’t tell	Yes	Yes	9
Stöckigt	Yes	Yes	Yes	Yes	Can’t tell	No	Yes	Can’t tell	Yes	Can’t tell	7.5
Ee et al. (36)	Yes	Yes	Yes	Yes	Yes	Can’t tell	Yes	Yes	Yes	Can’t tell	9
Eriksen et al. (37)	Yes	Yes	Yes	Yes	Can’t tell	No	Yes	Yes	Yes	Can’t tell	8
Hung et al. (44)	Yes	Yes	Yes	Yes	Yes	Yes	Yes	Yes	Yes	Can’t tell	9.5
Kweku Sey and Hunter (55)	Yes	Yes	Yes	Can’t tell	Yes	No	Can’t tell	Can’t tell	Yes	Can’t tell	7
Liu et al. (34)	Yes	Yes	Yes	Yes	Yes	No	Yes	Yes	Yes	Yes	9
Liu et al. (65)	Yes	Yes	Yes	Yes	Yes	No	Yes	Yes	Yes	Yes	9
Peter et al. (67)	Yes	Yes	Yes	Yes	Yes	No	Yes	Can’t tell	Yes	Yes	8.5
Mao et al. (49)	Yes	Yes	Yes	Yes	Yes	Can’t tell	No	No	Yes	Can’t tell	7
Murley et al. (43)	Yes	Yes	Yes	Yes	Yes	No	No	Yes	Yes	Yes	8
Oberoi et al. (40)	Yes	Yes	Yes	Yes	Yes	No	No	Yes	Yes	Yes	8
Osypiuk et al. (47)	Yes	Yes	Yes	Yes	Yes	No	Yes	Yes	Yes	Yes	9
Özdemir and Taşcı (46)	Yes	Yes	Yes	Yes	Yes	No	Yes	No	Can’t tell	Can’t tell	7
Mackereth and Stringer (32)	Yes	Yes	Yes	Yes	Yes	No	Yes	Yes	Yes	Yes	9
Porter et al. (41)	Yes	Yes	Yes	Yes	Yes	No	Can’t tell	Yes	Can’t tell	Yes	8
Price et al. (53)	Yes	Yes	Yes	Can’t tell	Yes	Can’t tell	Can’t tell	Yes	Yes	Can’t tell	8
Price et al. (58)	Yes	Yes	Yes	Yes	Yes	Can’t tell	Yes	Yes	Yes	Can’t tell	9
Romero et al. (38)	Yes	Yes	Yes	Yes	Can’t tell	No	Can’t tell	No	Can’t tell	Yes	6.5
Schapira et al. (61)	Yes	Yes	Yes	Yes	Yes	Can’t tell	Can’t tell	Can’t tell	Yes	Can’t tell	8
Walker et al. (50)	Yes	Yes	Yes	Yes	Yes	No	Yes	No	Can’t tell	Can’t tell	7
Wang et al. (30)	Yes	Yes	Yes	Can’t tell	Yes	No	Yes	Can’t tell	Yes	Can’t tell	7.5
Xu et al. (56)	Yes	Yes	Yes	Yes	Yes	No	Yes	Can’t tell	Yes	Can’t tell	8
Yu et al. (31)	Yes	Yes	Yes	Can’t tell	Yes	No	Yes	Yes	Yes	Can’t tell	8
Yu (59)	Yes	Yes	Yes	Yes	Yes	Yes	Yes	Yes	Yes	Yes	10
Hu et al. (57)	Yes	Yes	Yes	Yes	Yes	No	No	Can’t tell	Yes	Yes	7.5
McPherson et al. (68)	Yes	Yes	Yes	Yes	Yes	No	Can’t tell	Can’t tell	Can’t tell	Yes	7.5
Li et al. (51)	Yes	Yes	Yes	Yes	Yes	No	Yes	Can’t tell	Can’t tell	Can’t tell	7.5
Qin (62)	Yes	Yes	Yes	Yes	Yes	No	Yes	Yes	Can’t tell	Can’t tell	8
Song and Wang (54)	Yes	Yes	Yes	Yes	Yes	No	Yes	Can’t tell	Can’t tell	Can’t tell	7.5
Wang (63)	Yes	Yes	Yes	Yes	Yes	Can’t tell	Yes	Yes	Yes	Can’t tell	9
Xie	Yes	Yes	Yes	Yes	Yes	Can’t tell	Yes	Yes	Yes	Yes	9.5
Yin and Yu (66)	Yes	Yes	Yes	Yes	Yes	No	Yes	Yes	Yes	Can’t tell	8.5
Zhang (52)	Yes	Yes	Yes	Yes	Yes	Can’t tell	Yes	Can’t tell	Can’t tell	Can’t tell	8
Zhang et al. (64)	Yes	Yes	Yes	Can’t tell	Yes	Yes	Yes	Yes	Yes	Yes	9.5
Yes	39	39	39	33	36	5	28	19	30	18	–
No	0	0	0	0	0	24	4	6	0	0	–
Can’t tell	0	0	0	6	3	10	7	14	9	21	–

1. Was there a clear statement of the aim of the research? 2. Is a qualitative methodology appropriate? 3. Was the research design appropriate to address the aims of the research? 4. Was the recruitment strategy appropriate to the aims of the research? 5. Was the data collected in a way that addressed the research issue? 6. Has the relationship between researcher and participants been adequately considered? 7. Have ethical issues been taken into consideration? 8. Was the data analysis sufficiently rigorous? 9. Is there a clear statement of findings? 10. How valuable is the research?

### 3.3 Synthesis of findings

**FIGURE 3:**
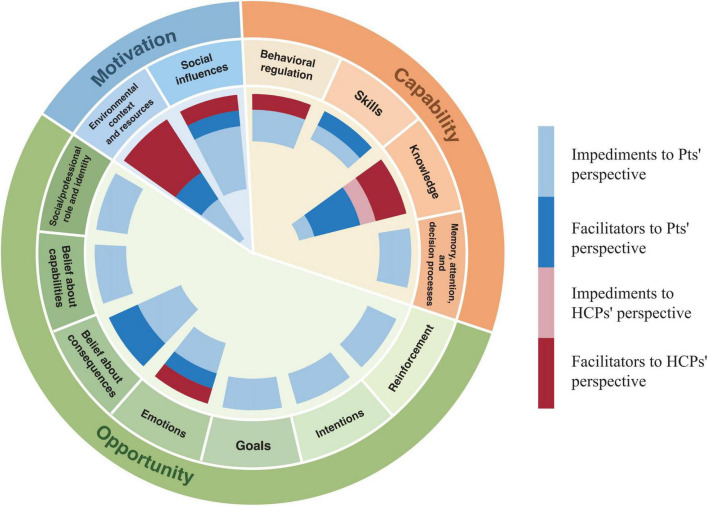
Frequency of impact factors mapped to Capability-Opportunity-Motivation-Behaviour (COM-B) and Theoretical Domains Framework (TDF) frameworks.

We summarized enablers and barriers to using TCM in cancer care from patients’ and HCPs’ perspectives, aligned with COM-B and TDF. Patients identified 28 facilitators and 10 barriers across capability, motivation, opportunity components, and 13 TDF domains. HCPs noted fewer factors: one facilitator and nine barriers. No new themes emerged that could not be categorized within the existing frameworks (Details in Figure 3 and the Supplementary Table 5).

#### 3.3.1 Capability

Capability is defined as the physical and mental abilities that affect the use of TCM by cancer patients and HCPs (28, 29). A total of 10 patients’ perspective themes based on the capability component were generated in this study, with six facilitating and four impeding themes, four HCPs’ perspective themes, one facilitating and three impeding themes.

For patients, the ability to understand and process information related to TCM and the self-management of health plays a critical role in facilitating behavioral choices. Specifically, patients can obtain information about TCM through multiple channels, including its benefits in enhancing the efficacy of conventional treatments (30, 31), reducing side effects (30–42), improving mental health (32, 33, 35, 39, 40, 42–45), enhancing well-being and coping (30, 32, 33, 35, 41–48). Through the evaluation and assessment of the above information, patients may be more likely to choose TCM as a supportive therapy in tumor treatment (30–34, 42, 49–51). Additionally, by practicing TCM self-management skills in daily life, such as acupressure (46, 51), auricular pressure (52), and dietary therapy (35), patients can more effectively manage health issues and improve treatment adherence, ultimately increasing the likelihood of choosing TCM therapies.

Moreover, TCM therapy, rooted in traditional Chinese culture, adopts a holistic perspective that views health as an interconnected whole and emphasizes balance in body, mind, and spirit (40, 41). While addressing physical discomfort, it also focuses on the regulation and management of emotions and stress (33, 50). Additionally, by fostering an understanding of healthy behaviors and lifestyle changes, TCM enhances patients’ health awareness and their sense of self-efficacy in pursuing a healthier lifestyle (41). This process strengthens their psychological capability and indirectly promotes the adoption of TCM treatments.

The barriers influencing patients’ decision to adopt TCM therapies can be mapped to two domains: knowledge and skills. In the knowledge domain, barriers include a lack of understanding of existing efficacy evidence, as well as doubts regarding the mechanism of action (53), effectiveness (30, 33, 38, 49, 50), duration of therapeutic benefits, and safety of the therapies (42). Furthermore, information asymmetry between patients and healthcare providers is another potential barrier, primarily reflected in insufficient patient education by doctors regarding treatment frequency and precautions (36, 38, 48, 51, 54). In the domain of skills, patients found it challenging to locate TCM physicians with oncology expertise, which hinders their choices (55).

From HCP’s perspective, the potential factors associated with capability can be mapped to the knowledge and behavioral modification domains. In terms of knowledge, recognizing the benefits of TCM as a supportive therapy in clinical practice can encourage behavioral choices (32, 53, 55–59). Conversely insufficient evidence on efficacy, a lack of evidence-based data, and limited access to reliable information significantly hinder behavioral choices (32, 55). Behavioral regulation-related impediments involve a lack of interdisciplinary communication (32), which greatly hampers the likelihood that oncologists and physicians will be able to understand the mechanisms, and effectiveness of TCM.

### 3.3.2 Motivation

Motivation refers to the cognitive processes that stimulate and guide the selection of TCM in cancer care (28). It can be categorized as reflective and automatic motivation, which can map to multiple domains in the TDF (28, 29, 60). A total of 18 patients’ perspective themes were generated in this study, with 16 facilitating themes and three hindering themes; one hindering theme from the HCPs’ perspective was related to the domain of emotions, and no relevant facilitating themes were found.

Key factors influencing patients’ choice of TCM include positive expectations of its supportive therapeutic effects (33–35, 39–42, 44, 50, 53, 54, 56), negative emotions regarding the side effects of conventional treatments (31, 33, 39, 44, 47, 49, 61, 62), emotional distress related to the disease (30, 40, 42, 44, 47, 50, 55, 59, 63), the pursuit of integrated TCM and conventional therapies (31, 33, 39, 41, 42, 44, 51, 55, 56, 61–64), and behavioral reinforcement from positive treatment experience (40, 46, 53, 62, 63). Additionally, cultural beliefs (31, 48, 51, 56, 63, 65), trust in the authority of practitioners (34, 62, 66), and the desire for a safer medical environment in resource-limited settings (65) play significant roles. Notably, in terms of intention, minority of patients view TCM as a potential alternative therapy, believing it can directly treat cancer by reducing tumor size, with some even claiming it cured their primary or metastatic lesions (34, 56, 63). However, such subjective views should be interpreted with caution, as they lack robust clinical evidence and may be influenced by individual experiences or cognitive bias. Finally, patients’ pursuit of control over their illness and a desire for self-help further drive the choice of TCM in cancer care (41, 44, 55).

Factors limiting patients’ choice of TCM include distrust in its treatment outcomes (32, 37, 38, 42, 51, 54, 65), and concerns about the quality and safety of the products associated with herbal medicines (35, 42, 44, 48). Negative emotions associated with the treatment process, such as needle phobia (32, 33, 36), shame from body exposure (33, 40), nausea and discomfort after consuming herbal medicines (31, 33, 35, 50), and fear triggered by the treatment environment (33), decrease the likelihood of patients choosing TCM.

Under motivation, the main barrier to HCP behavior is patients’ discomfort with TCM use (59).

#### 3.3.3 Opportunity

Opportunity refers to external factors influencing the use of TCM in cancer care by patients and HCPs, categorized into social and physical opportunities (28). A total of 10 patients’ perspective themes based on the opportunity component were generated in this study, including seven facilitating themes, three hindering themes, and five themes from the HCPs’ perspective, all hindering factors.

From the patients’ perspective, key facilitators within the social influence cluster include positive influences from significant others, such as family members, colleagues, and doctors (30, 32, 35, 41, 42, 44, 49, 51, 52, 56, 62–65), the media (32, 49, 63, 64), and ward-mates (40, 43, 47, 50, 56, 67). Studies indicate that patients immersed in positive doctor-patient relationships, such as through listening (30, 32, 40, 41, 43, 45, 50, 51, 62, 64, 66), encouragement (39), and support (32, 34, 39, 44, 50, 51, 62, 67), play an important role in decision-making behaviors. Facilitating factors related to environmental context and resources include a comfortable treatment environment (40, 45), the authority of the clinic environment (65), economic feasibility and policy support (31, 34, 36, 41, 55, 56) (e.g., health insurance, health care policy, etc.). Three potential barriers include oncologists’ negative attitudes toward TCM (36, 41, 42, 44, 56, 62), false advertising (30), and inadequate medical resources (e.g., high treatment and transportation costs, long treatment duration, and excessive waiting times) (30, 32, 33, 36, 40–42, 44, 49, 52, 54–56, 59, 61, 63, 66).

From the perspective of healthcare professionals (HCPs), similar to patients’ views, the main barriers include oncologists’ negative attitudes toward TCM (32, 56) and the limited healthcare budgets (55, 56). Furthermore, TCM experts emphasized that the absence of cross-disciplinary communication with oncologists impedes their comprehension of the efficacy and safety of TCM (32), thereby indirectly influencing the adoption of TCM treatments.

#### 3.3.4 Subgroup analysis based on TCM treatment modalities

This subgroup analysis of eight TCM therapies shows that the overall results remain robust despite minor variations due to intervention characteristics, with three facilitating factors and six barriers varying across interventions (Supplementary Table 6).

Among facilitating factors, HCPs and patients recognize TCM’s role in supportive cancer care and its position as an adjunct therapy. Some doctors also use anticancer herbs to enhance supportive effects, such as inhibiting tumor growth and metastasis (68). In contrast, some patients may view herbal therapy as an alternative to conventional treatment (34, 63). TCM interventions with self-care characteristics exhibit distinct advantages: acupressure is simple to learn and easy to apply (46); non-prescribed functional herbal products are available in various forms (e.g., tablets, teas, foods) and are easily accessible (35); dynamic therapies in mind-body practices (e.g., Qigong, Tai Chi) enhance physical function at appropriate intensity levels (35).

Regarding barriers, different TCM models present unique challenges. Regarding the perception of TCM efficacy and safety, some patients consider acupuncture may stimulate tumor growth (56) and are uncertain about its long-term efficacy (37, 38). In herbal therapy, both doctors and patients express concerns about the potential risks of herb-drug interactions (48, 68). In terms of economic burden, acupuncture increases costs due to its treatment frequency and duration (40, 61). In comparison, the high prices of standard herbal products, such as *Lingzhi* (Ganoderma lucidum) powder, add to the financial strain (44, 56). Negative experiences with TCM include needle phobia (32, 33, 36), and body exposure (39, 58) in acupuncture, pain and discomfort in acupressure (46, 51), unpleasant taste in herbal medicine and supplements (35, 63), and noise perception in Five Elements music therapy within mind-body therapies (31). Furthermore, unique barriers to non-prescribed functional herbal products include deceptive advertising (30), concerns about product transparency (35, 48), and lack of usage guidance (35, 48, 51).

Limited research on herbal footbath and moxibustion identified no distinct facilitating or barrier factors for these modalities. Therefore, this subgroup analysis highlights the diverse characteristics of TCM interventions in supportive cancer care, emphasizing their potential benefits and challenges in clinical application.

## 4 Discussion

This study systematically reviewed 39 qualitative studies exploring using single or multiple TCM therapies as supportive care in cancer management. As such, we analyzed the enablers and barriers influencing healthcare providers’ and patients’ choices of TCM. Although guidelines and clinical research supporting TCM as a complementary therapy in oncology have increased in recent years, its implementation barriers and practical challenges in real-world clinical settings remain underexplored. To our knowledge, this is the first systematic review to specifically address the implementation factors influencing the use of TCM in oncology. This study also represents a critical first step in bridging this pervasive implementation gap. Furthermore, we propose a series of theory-based behavioral interventions to address these challenges. Importantly, the factors and interventions identified in this study can guide clinicians and policymakers in adapting integrated management strategies and allocating resources to enhance the well-being of cancer patients.

### 4.1 Main finding

#### 4.1.1 Geographical differences in TCM oncology research

From the included studies, Western countries focus more on non-pharmacological TCM therapies such as acupuncture, Qigong, and massage. In contrast, research in Asian countries, particularly China, is more diverse, significantly emphasizing herbal treatment. This disparity is closely linked to the cultural and healthcare system differences between East and West. For example, acupuncture has been integrated into the healthcare systems of some Western countries (69). However, due to strict Good Manufacturing Practice (GMP) regulations in Western countries (70), issues related to the quality, purity, and standardization of herbal medicine remain unresolved. Consequently, research in the West often centers on single herbs and their active components, resulting in lower clinical use and research frequency compared to Asian countries. Unfortunately, our studies include participants from diverse age groups and disease backgrounds, challenging traditional stereotypes of TCM users and indicating broader cultural acceptance (71).

#### 4.1.2 Cognitive and evidence gaps in TCM oncology practice

Our study indicates that the lack of evidence-based data and the cognitive gap between HCPs and patients are significant barriers to integrating TCM into cancer care. This aligns with challenges commonly observed in implementing other complementary and alternative therapies (72).

Evidence gaps are primarily reflected in the following areas: (1) Lack of research and standardized assessment of symptom clusters: Cancer patients often experience interrelated symptoms, collectively referred to as “symptom clusters,” sharing common mechanisms or pathways (44). Multiple studies included in this research suggest that TCM offers effective interventions for managing symptom clusters (53). However, standardized methods for measuring these clusters remain lacking. Future studies should focus on developing precise and consistent outcome measures to better evaluate the dynamic changes and treatment effects of symptom clusters (73); (2) Incomplete research on herbal toxicity and safety: There are notable differences in how healthcare professionals perceive the safety of herbal medicines. Oncologists, in particular, express caution regarding potential herb-drug interactions (HDIs) when herbal treatments are combined with chemotherapy or other conventional therapies (74). As such, they often cite concerns about compromised efficacy or increased toxicity. Moreover, the hepatic and renal toxicity of herbal medicines requires further investigation (75); (3) Insufficient clinical evidence for direct anti-tumor effects of herbal medicines: Although some TCM practitioners use specific herbal medicines for their purported anticancer effects (68), laboratory and preclinical studies have demonstrated anti-tumor activity in certain herbs (e.g., Astragalus) (13). However, high-quality RCTs are lacking to validate their clinical efficacy and identify appropriate patient populations; (4) Limited evidence on the long-term effectiveness of interventions: Subgroup analyses reveal that some patients remain skeptical about the long-term efficacy of acupuncture (38). Consequently, current evidence on the sustained benefits of acupuncture in cancer management is inconsistent (4). Future research should prioritize well-designed, long-term RCTs to provide robust evidence for their long-term efficacy.

The cognitive gap is primarily reflected in limited knowledge of key areas, including cancer biology, the scope and efficacy of TCM treatments, mechanisms of action, safety, and the optimal timing for integration into conventional cancer care. From the perspective of HCPs, concerns include doubts about the quality of evidence, a disconnect between research findings and clinical practice, and cross-cultural differences in understanding. Additionally, TCM practitioners may lack expertise in oncology and herbal pharmacology. For instance, some rely on traditional knowledge and clinical experience to prescribe herbs, such as using phytoestrogen-containing Yin-tonifying herbs for estrogen-positive breast cancer patients, as reported in certain studies (68).

Despite systematic reviews supporting the efficacy and safety of TCM in IO, the translating this evidence into practice faces several barriers. These include: (1) Trial design based on syndrome differentiation: TCM emphasizes individualized treatment based on syndrome differentiation, which introduces subjectivity in defining inclusion criteria, intervention, and outcome measures in RCTs (76). While this approach highlights TCM’s unique ability to address patients’ symptoms and conditions dynamically, it also demands higher standards of training and quality control, potentially compromising the reliability and clinical applicability of results; (2) Limited representativeness of populations: Most TCM research is conducted in East Asia, with culturally homogeneous populations, limiting the generalizability of findings to diverse settings and populations; and (3) Lack of effective evidence network collaboration: Evidence networks play an important role in connecting researchers, policymakers, and practitioners to facilitate the generation, dissemination, and application of evidence to inform decisions (77). However, the absence of a structured evidence network in the TCM field hinders the global dissemination and application of TCM research findings.

#### 4.1.3 Synergistic models and challenges in integrative cancer care

In Integrative Cancer Care (ICC), combining biomedical treatments with TCM demonstrates notable complementary strengths. Biomedical treatments are disease-centered care, focusing on tumor control and clinical outcomes. In contrast, TCM adopts patient-centered care, prioritizing holistic health, psychological support, and quality of life. Thus, this synergistic model provides a valuable framework for optimizing cancer care and improving patient outcomes, quality of life, and overall care experiences (78).

In patient-centered care models, Shared Decision Making (SDM) has garnered increasing attention. SDM emphasizes providing evidence-based, comprehensive recommendations while respecting patients’ preferences, beliefs, and priorities, enabling healthcare professionals and patients to collaboratively develop personalized treatment plans (79). This approach not only reduces decision-making conflicts but also enhances patients’ engagement and trust in their treatment. Our study found that the application of SDM demonstrates significant advantages in real-world TCM clinical practice. The “patient-centered” philosophy of TCM aligns closely with the core objectives of SDM, fostering a sense of involvement in decision-making (80). For instance, prioritizing patients’ most pressing symptoms and incorporating their preferences in selecting herbal formulations further underscores TCM’s personalized approach, improving the overall care experience.

Moreover, our research indicated that in this patient-centered model, patients often report that a supportive environment, active listening by doctors, and therapeutic alliance play an important role in psychological well-being. This relationship improves mental health and promotes holistic recovery, enhancing the quality of care (32, 51). Interestingly, from the physicians’ perspective, trust-based patient-provider relationships and patient-centered communication are tools to encourage patients to express their needs and disclose health issues (58). Like other chronic disease management settings, both doctors and patients emphasize the importance of building long-term trust to improve clinical outcomes and ensure adequate care (81).

However, despite these benefits, integrating TCM into integrative cancer care poses challenges, mainly due to differences in treatment models and the lack of interdisciplinary communication. Notably, some patients in our included studies reported withholding information about their use of TCM due to perceived negative attitudes from physicians. Studies indicate that approximately 29.3% of patients in the United States do not disclose their use of complementary and alternative medicine (CAM) to their doctors (12). This lack of communication hinders the proper integration of TCM with conventional therapies and negatively impacts optimal medical decision-making.

### 4.2 Potential interventions to promote TCM implementation

To advance the integration of TCM into IO services, this study identified potential strategies to address the implementation barriers. These barriers were mapped to eight of the 14 TDF domains, highlighting the multifaceted nature of obstacles to TCM adoption. Using the Behavior Change Wheel (BCW) (82) and Behavior Change Techniques (BCTs) (60), targeted interventions were designed to address key implementation challenges, as detailed in Table 3.

**TABLE 3:** Recommended behavior change intervention.

Implementation issues for improvement	Target population	COM-B component—TDF domain	Intervention function	Policy category	Behavior change technique(s)	Behavior changes
Lack of knowledge about TCM in supportive cancer care	Patients, Biomedical healthcare providers	Capability—Knowledge, Motivation—Belief about consequences	Education	Communication/marketing	Health consequences	Dissemination of knowledge on the benefits, safety, and therapeutic scope of TCM
Lack of availability and utilization of information on TCM practice	Biomedical healthcare providers	Capability—Knowledge, Motivation—Emotions, Opportunity—Social influences	Enablement	Regulation	Instruction on how to perform a behavior	Development and use of tools for identifying and assessing TCM use
	Healthcare providers	Capability—Knowledge	Environmental restructuring	Guidelines	Instruction on how to perform a behavior	More clinical studies to supplement evidence for guideline gaps
	Policy formulators and implementers	Capability—Knowledge, Behavioral regulation, Motivation—Emotions, Opportunity—Social influences	Environmental restructuring	Environmental/ social planning	Restructuring the physical environment	Use implementation science theories/frameworks to identify barriers and assign follow-up interventions
Lack of multidisciplinary collaborative environment	Healthcare providers	Capability—Behavioral regulation	Environmental restructuring	Communication/ marketing	Restructuring the physical environment	Regularized multidisciplinary interaction opportunities and integration of TCM courses into medical education
The doctor-patient information gap ignored in communication	TCM practitioners	Capability—Knowledge	Restriction	Guidelines	Instruction on how to perform a behavior	Designated communication guidelines handbook or tools
Limited availability of specialists	Policy formulators and implementers	Opportunity—Environmental context and resources	Environmental restructuring	Environmental/ social planning	Social support (practical)	Improvement of structured referral pathways and delivery systems
	TCM practitioners	Capability—Skills	Training	Communication/ marketing	Behavioral practice/ rehearsal	Training of TCM practitioners in specialized oncology knowledge
	Patients	Opportunity—Environmental context and resources	Enablement	Environmental/ social planning	Social support (practical)	Building a social environment for self-help
Safety risks of herbal dietary supplements	Policy formulators and implementers	Motivation—Belief about consequences Opportunity—Environmental context and resources	Restriction	Legislation	Punishment	Designation of whole regulatory policies and penalization mechanisms
Financial barriers to TCM implementation	Policy formulators and implementers	Opportunity—Environmental context and resources	Environmental restructuring	Fiscal	Restructuring the social environment	Assessing health care needs and integrating adjustments to financial support

#### 4.2.1 Disseminating knowledge of TCM in supportive cancer care

Through the “education” intervention function, evidence-based knowledge, guidelines, and research on TCM can be disseminated via various media channels, including online learning platforms, patient guidelines, educational brochures, and courses (83). Importantly users should critically evaluate online information, as research shows most online discussions about CAM are commercially and potentially misleading (84).

#### 4.2.2 Addressing evidence and utilization gaps

To overcome the challenge of limited evidence and low utilization of TCM guidelines, we propose systematic improvements through the intervention functions of “Enablement” and “Environmental restructuring.” First, conducting more high-quality TCM clinical trials is essential to supplement existing evidence for clinical practice. Second, standardized TCM utilization and risk management tools should be developed, drawing on examples such as the questionnaire tool by Shalgouny et al. (72). Finally, existing guideline implementation frameworks [e.g., Knowledge-to-Action framework (85), Guideline Implementability Decision Excellence Model (86)] can be utilized to systematically analyze barriers in diverse implementation contexts and design tailored solutions.

#### 4.2.3 Promotion of evidence-based collaborative care

Establishing a multidisciplinary environment is crucial for integrating TCM into oncology care. This includes adopting the Collaborative Care Management (CoCM) model, organizing regular case discussions, and incorporating CAM education into medical training to enhance understanding and acceptance of TCM.

#### 4.2.4 Addressing communication gaps

Utilizing patient-directed knowledge tools to address information gaps in doctor-patient interactions. For example, using a structured checklist (87) to address key patient concerns for more efficient doctor-patient communication, and creating informational leaflets (88) to provide guidance on treatment timelines, efficacy, and dietary therapies.

#### 4.2.5 Improving access to TCM specialists

To address the shortage of TCM specialists, interventions should incorporate Environmental Restructuring, Training, and Enablement. Environmental Restructuring can be achieved by developing structured referral pathways that integrate TCM services into existing oncology care workflows. For example, oncology clinics could implement a standardized checklist or decision-support tool embedded within electronic health records (EHR) to streamline referrals to TCM practitioners. Additionally, creating formal partnerships between oncology centers and TCM clinics would facilitate coordinated care (89). Training involves training TCM practitioners in specialized oncology knowledge, such as cancer biology, pharmacology, and the management of treatment-related side effects. Enablement focuses on fostering patient-centered initiatives, such as establishing community-based Tai Chi classes and training patients in auricular acupuncture self-care, to empower individuals in managing their symptoms and improving quality of life.

#### 4.2.6 Regulating herbal supplements

To reduce safety risks, regional and cultural differences will be considered to formulate strict laws and regulations to restrict misleading advertisements of herbal dietary supplements and improve the transparency of production.

#### 4.2.7 Financial barriers

As the cost of TCM-related products and services is high, financial barriers may impact TCM implementation more (90). To address financial barriers impacting TCM implementation, it is necessary to assess healthcare needs and adjust financial support while considering variations in healthcare policies and payment models across countries.

As we identified impediments mostly at the systemic level, some of the structural changes proposed are complicated. Future research should focus on developing targeted interventions and implementation strategies. Furthermore, the research could be refined through interviews with relevant stakeholders to adapt to the specific contexts of different countries.

### 4.3 Strengths and limitations

The primary strength of this study lies in its innovative approach to examining attitudes toward the application of TCM in oncology from both patient and healthcare provider perspectives. By using established theoretical frameworks (TDF and COM-B), the study systematically identifies key factors influencing the implementation of TCM. Moreover, it applies the BCW and BCT techniques to propose potential solutions for advancing integrative oncology care. As a key component of TCI, the findings of this study not only complement existing research on implementation factors but also uncover previously underexplored barriers (91), such as misleading advertising and communication gaps.

Moreover, the factors and strategies identified in this study are not limited to TCM but may also be broadly applicable to other complementary medicine practices in oncology care (92). These practices similarly face challenges such as insufficient interdisciplinary collaboration, a lack of trust between patients and providers, and evidence gaps. Like TCM, their implementation may also be constrained by cultural acceptance, the adequacy of healthcare infrastructure, and the availability of trained professionals.

This meta-synthesis has several recognized limitations. Most included studies were conducted in single regions or institutions, limiting the representativeness of the findings and the ability to explore how cultural and geographical differences affect TCM implementation. Consequently, this also hindered detailed subgroup analyses by profession, ethnicity, or knowledge level. Moreover, some studies span over 15 years, during which the context of TCM practices may have undergone significant changes, such as shifts in research priorities for intervention models and adjustments to insurance coverage. These changes could impact the generalizability of the findings and their relevance to current clinical settings. Overlaps between certain TDF domains were also identified, complicating factor classification and intervention development, highlighting the need for framework refinement. Finally, the proposed interventions lack empirical validation, requiring further research to assess their real-world effectiveness.

### 4.4 Implication for policymakers

The efficacy of TCM in supportive cancer care is supported by evidence; however, as a complex intervention, its outcomes are influenced by multiple variables, such as treatment dosage, practitioner expertise, and cultural context (93). As such, these factors place higher demands on its clinical translation and implementation. Therefore, this study identifies key influencing factors and proposes potential interventions to facilitate the effective integration of TCM into clinical practice.

Policymakers should first assess the suitability of TCM within local healthcare systems and its cross-cultural applicability. Based on these evaluations, recommended policy measures include optimizing healthcare policies and funding, strengthening TCM education and training for healthcare providers, establishing efficient TCM referral systems, and enhancing the safety regulation of herbal and dietary supplements. Additionally, increasing funding for TCM cancer research, particularly for empirical studies on intervention strategies and improvements, is essential to provide scientific evidence for its integration into oncology care. At the same time, efforts should focus on advancing multi-center and cross-regional RCTs to generate robust evidence reflecting TCM’s effectiveness across diverse populations, healthcare systems, and cultural contexts, thereby enhancing its global applicability in oncology.

## 5 Conclusion

Traditional Chinese Medicine plays an important role in integrative oncology care, and patients’ interest in complementary therapy has grown. However, several complex challenges hinder its implementation. Our study identifies key behavioral determinants affecting TCM use in oncology using the COM-B model and TDF framework. Knowledge gaps, lack of multidisciplinary communication, low number of practitioners with specialist knowledge, and lack of an economically supportive environment are the main impeding factors. Guided by BCW theory and BCT technology, we propose several generalizable intervention functions for the above impediments, with the expectation that they will provide a foundation for filling the practice gap of TCM in oncology care. Future research should also first assess the suitability of TCM in cancer care based on local clinical evidence, identifying appropriate interventions and their implementation strategies. Building on this, targeted interventions should be evaluated and implemented across diverse healthcare systems, accompanied by localized empirical studies to further enhance the integration of TCM into clinical oncology practice.

## Data Availability

The original contributions presented in this study are included in this article/Supplementary material, further inquiries can be directed to the corresponding authors.
