# Supplementary material for: Factors influencing the utilization of Traditional Chinese Medicine in cancer treatment: a qualitative meta-synthesis of patient and healthcare professional perspectives
Source: Front Med (Lausanne). 2025 Mar 5;12:1501918. doi: 10.3389/fmed.2025.1501918 (PMC11920129; doi:10.3389/fmed.2025.1501918)
Supplement: Supplementary file 1 [file Table_1.docx]

**Supplementary Files**

Table S1. PRISMA checklist

| **Section and Topic** | **Item #** | **Checklist item** | **Location where item is reported** |
| --- | --- | --- | --- |
| **TITLE** | | |  |
| Title | 1 | Identify the report as a systematic review. | 1 |
| **ABSTRACT** | | |  |
| Abstract | 2 | See the PRISMA 2020 for Abstracts checklist. | 1-3 |
| **INTRODUCTION** | | |  |
| Rationale | 3 | Describe the rationale for the review in the context of existing knowledge. | 2,3 |
| Objectives | 4 | Provide an explicit statement of the objective(s) or question(s) the review addresses. | 2,3 |
| **METHODS** | | |  |
| Eligibility criteria | 5 | Specify the inclusion and exclusion criteria for the review and how studies were grouped for the syntheses. | 4 |
| Information sources | 6 | Specify all databases, registers, websites, organisations, reference lists and other sources searched or consulted to identify studies. Specify the date when each source was last searched or consulted. | 4,5 |
| Search strategy | 7 | Present the full search strategies for all databases, registers and websites, including any filters and limits used. | 3,4; Supplementary  Table S2 |
| Selection process | 8 | Specify the methods used to decide whether a study met the inclusion criteria of the review, including how many reviewers screened each record and each report retrieved, whether they worked independently, and if applicable, details of automation tools used in the process. | 6,7 |
| Data collection process | 9 | Specify the methods used to collect data from reports, including how many reviewers collected data from each report, whether they worked independently, any processes for obtaining or confirming data from study investigators, and if applicable, details of automation tools used in the process. | 5-7 |
| Data items | 10a | List and define all outcomes for which data were sought. Specify whether all results that were compatible with each outcome domain in each study were sought (e.g. for all measures, time points, analyses), and if not, the methods used to decide which results to collect. | 5,18 |
|  | 10b | List and define all other variables for which data were sought (e.g. participant and intervention characteristics, funding sources). Describe any assumptions made about any missing or unclear information. | 5,18 |
| Study risk of bias assessment | 11 | Specify the methods used to assess risk of bias in the included studies, including details of the tool(s) used, how many reviewers assessed each study and whether they worked independently, and if applicable, details of automation tools used in the process. | 16-18 |
| Effect measures | 12 | Specify for each outcome the effect measure(s) (e.g. risk ratio, mean difference) used in the synthesis or presentation of results. | None |
| Synthesis methods | 13a | Describe the processes used to decide which studies were eligible for each synthesis (e.g. tabulating the study intervention characteristics and comparing against the planned groups for each synthesis (item #5)). | 5,6 |
|  | 13b | Describe any methods required to prepare the data for presentation or synthesis, such as handling of missing summary statistics, or data conversions. | 5,6 |
|  | 13c | Describe any methods used to tabulate or visually display results of individual studies and syntheses. | 5,6 |
|  | 13d | Describe any methods used to synthesize results and provide a rationale for the choice(s). If meta-analysis was performed, describe the model(s), method(s) to identify the presence and extent of statistical heterogeneity, and software package(s) used. | 5,6 |
|  | 13e | Describe any methods used to explore possible causes of heterogeneity among study results (e.g. subgroup analysis, meta-regression). | None |
|  | 13f | Describe any sensitivity analyses conducted to assess robustness of the synthesized results. | None |
| Reporting bias assessment | 14 | Describe any methods used to assess risk of bias due to missing results in a synthesis (arising from reporting biases). | None |
| Certainty assessment | 15 | Describe any methods used to assess certainty (or confidence) in the body of evidence for an outcome. | None |
| **RESULTS** | | |  |
| Study selection | 16a | Describe the results of the search and selection process, from the number of records identified in the search to the number of studies included in the review, ideally using a flow diagram. | 6,7, fig 1 |
|  | 16b | Cite studies that might appear to meet the inclusion criteria, but which were excluded, and explain why they were excluded. | 7 |
| Study characteristics | 17 | Cite each included study and present its characteristics. | 6-15, table 2 |
| Risk of bias in studies | 18 | Present assessments of risk of bias for each included study. | 16-18, table 3 |
| Results of individual studies | 19 | For all outcomes, present, for each study: (a) summary statistics for each group (where appropriate) and (b) an effect estimate and its precision (e.g. confidence/credible interval), ideally using structured tables or plots. | 18-27, fig 3 |
| Results of syntheses | 20a | For each synthesis, briefly summarise the characteristics and risk of bias among contributing studies. | 18-27, fig 3, table 4 |
|  | 20b | Present results of all statistical syntheses conducted. If meta-analysis was done, present for each the summary estimate and its precision (e.g. confidence/credible interval) and measures of statistical heterogeneity. If comparing groups, describe the direction of the effect. | 18-27, fig 3, table 4 |
|  | 20c | Present results of all investigations of possible causes of heterogeneity among study results. | None |
|  | 20d | Present results of all sensitivity analyses conducted to assess the robustness of the synthesized results. | None |
| Reporting biases | 21 | Present assessments of risk of bias due to missing results (arising from reporting biases) for each synthesis assessed. | None |
| Certainty of evidence | 22 | Present assessments of certainty (or confidence) in the body of evidence for each outcome assessed. | None |
| **DISCUSSION** | | |  |
| Discussion | 23a | Provide a general interpretation of the results in the context of other evidence. | 27-33 |
|  | 23b | Discuss any limitations of the evidence included in the review. | 34-35 |
|  | 23c | Discuss any limitations of the review processes used. | 34-35 |
|  | 23d | Discuss implications of the results for practice, policy, and future research. | 30-35 |
| **OTHER INFORMATION** | | |  |
| Registration and protocol | 24a | Provide registration information for the review, including register name and registration number, or state that the review was not registered. | 3 |
|  | 24b | Indicate where the review protocol can be accessed, or state that a protocol was not prepared. | 3 |
|  | 24c | Describe and explain any amendments to information provided at registration or in the protocol. | 3 |
| Support | 25 | Describe sources of financial or non-financial support for the review, and the role of the funders or sponsors in the review. | 36 |
| Competing interests | 26 | Declare any competing interests of review authors. | 36 |
| Availability of data, code and other materials | 27 | Report which of the following are publicly available and where they can be found: template data collection forms; data extracted from included studies; data used for all analyses; analytic code; any other materials used in the review. | 36 |

*From:* Page MJ, McKenzie JE, Bossuyt PM, Boutron I, Hoffmann TC, Mulrow CD, et al. The PRISMA 2020 statement: an updated guideline for reporting systematic reviews. BMJ 2021;372:n71. doi: 10.1136/bmj.n71

For more information, visit: <http://www.prisma-statement.org/>

Table S2. Search Strategy

| **Cochrane** | #1"Acupuncture":ti,ab,kw OR "Acupuncture Therapy":ti,ab,kw OR "acupuncture, ear":ti,ab,kw OR "Acupuncture Points":ti,ab,kw OR "Acupuncture Analgesia":ti,ab,kw OR "Electroacupuncture":ti,ab,kw OR "Transcutaneous Electric Nerve Stimulation":ti,ab,kw OR "Acupuncture":ti,ab,kw OR "Electroacupuncture":ti,ab,kw OR "body needling":ti,ab,kw OR "Acupuncture Therapy":ti,ab,kw OR "ear acupuncture":ti,ab,kw OR "auricular needling":ti,ab,kw OR "scalp acupuncture":ti,ab,kw OR "laser acupuncture":ti,ab,kw OR "transcutaneous electrical nerve stimulation":ti,ab,kw OR "moxibustion":ti,ab,kw OR "moxibustion":ti,ab,kw OR "moxa":ti,ab,kw OR "massage":ti,ab,kw OR "acupressure":ti,ab,kw OR "reflexotherapy":ti,ab,kw OR "medicine, ayurvedic":ti,ab,kw OR "aromatherapy":ti,ab,kw OR "Reflexology":ti,ab,kw OR "Rolfing":ti,ab,kw OR "Manual Therapies":ti,ab,kw OR "Manual Therapy":ti,ab,kw OR "therapies manual":ti,ab,kw OR "therapy manual":ti,ab,kw OR "Zone Therapy":ti,ab,kw OR "Zone Therapies":ti,ab,kw OR "therapy zone":ti,ab,kw OR "Massage Therapy":ti,ab,kw OR "Massage Therapies":ti,ab,kw OR "therapies massage":ti,ab,kw OR "therapy massage":ti,ab,kw OR "Shiatsu":ti,ab,kw OR "Zhi Ya":ti,ab,kw  #2“Medicine, Chinese Traditional”:ti,ab,kw OR “Medicine, Traditional”:ti,ab,kw OR “Complementary Therapies”:ti,ab,kw OR “Medicine, Complementary”:ti,ab,kw OR “Alternative Medicine”:ti,ab,kw OR “Drugs, Chinese Herbal”:ti,ab,kw OR “herb*”:ti,ab,kw OR “phytotherapy”:ti,ab,kw OR “complimentary next therap*”:ti,ab,kw OR decoction:ti,ab,kw OR granule*:ti,ab,kw OR pill:ti,ab,kw OR pills:ti,ab,kw OR tablet*:ti,ab,kw OR “traditional CHM” OR “herbal medicine” OR “integrative medicine” OR “Chinese herb” OR “Chinese patent medicine”  #3 #1 OR #2  #4“mixed methods”:ti,ab,kw OR "mixed study":ti,ab,kw OR "mixed research":ti,ab,kw OR "qualitative research":ti,ab,kw OR "qualitative study":ti,ab,kw OR "qualitative methodology":ti,ab,kw OR "phenomen* research":ti,ab,kw OR "grounded theory*":ti,ab,kw OR "ethnograph*":ti,ab,kw OR "historic research":ti,ab,kw OR "historic study":ti,ab,kw OR "action research":ti,ab,kw OR “focus group”:ti,ab,kw OR “narrative”:ti,ab,kw OR “lived experience*”:ti,ab,kw  #5 "neoplas*" OR “tumor*” OR "tumour" OR “krebs*”:ti,ab,kw OR “cancer*”:ti,ab,kw OR “malignan*”:ti,ab,kw OR “carcino*”:ti,ab,kw OR “karzino*”:ti,ab,kw OR “karzinom*”:ti,ab,kw OR “sarcom*”:ti,ab,kw OR “leukem*”:ti,ab,kw OR “leukaem*”:ti,ab,kw OR “leucem*”:ti,ab,kw OR “lymphom*”:ti,ab,kw OR “melano*”:ti,ab,kw OR “metastas*”:ti,ab,kw OR “mesothelio*”:ti,ab,kw OR “mesotelio*”:ti,ab,kw OR “carcinomatos*”:ti,ab,kw OR “gliom*”:ti,ab,kw OR “glioblastom*”:ti,ab,kw OR “osteo?sarcom*”:ti,ab,kw OR “blastom*”:ti,ab,kw OR “neuroblastom*”:ti,ab,kw OR oncology:ti,ab,kw  #6 #3 AND #4 AND #5 |
| --- | --- |
| **PubMed** | #1“Medicine, Chinese Traditional” [MeSH Terms] OR “Medicine, Traditional” [MeSH Terms] OR “Complementary Therapies” [MeSH Terms] OR “Medicine, Complementary” [MeSH Terms] OR “Alternative Medicine” [MeSH Terms] OR “Drugs, Chinese Herbal” [MeSH Terms] OR “herb*” [Title/Abstract] OR “phytotherapy” [Title/Abstract] OR “complimentary next therap*” [Title/Abstract] OR decoction[Title/Abstract] OR granule*[Title/Abstract] OR pill[Title/Abstract] OR pills[Title/Abstract] OR tablet*[Title/Abstract] OR “traditional CHM” OR “herbal medicine” OR “integrative medicine” OR “Chinese herb” OR “Chinese patent medicine”  #2"Acupuncture"[MeSH Terms] OR "Acupuncture Therapy"[MeSH Terms] OR "acupuncture, ear"[MeSH Terms] OR "Acupuncture Points"[MeSH Terms] OR "Acupuncture Analgesia"[MeSH Terms] OR "Electroacupuncture"[MeSH Terms] OR "Transcutaneous Electric Nerve Stimulation"[MeSH Terms] OR "Acupuncture"[Title/Abstract] OR "Electroacupuncture"[Title/Abstract] OR "body needling"[Title/Abstract] OR "Acupuncture Therapy"[Title/Abstract] OR "ear acupuncture"[Title/Abstract] OR "auricular needling"[Title/Abstract] OR "scalp acupuncture"[Title/Abstract] OR "laser acupuncture"[Title/Abstract] OR "transcutaneous electrical nerve stimulation"[Title/Abstract] OR "moxibustion"[MeSH Terms] OR "moxibustion"[Title/Abstract] OR "moxa"[Title/Abstract] OR "massage"[MeSH Terms] OR "acupressure"[MeSH Terms] OR "reflexotherapy"[MeSH Terms] OR "medicine, ayurvedic"[MeSH Terms] OR "aromatherapy"[MeSH Terms] OR "Reflexology"[Title/Abstract] OR "Rolfing"[Title/Abstract] OR "Manual Therapies"[Title/Abstract] OR "Manual Therapy"[Title/Abstract] OR "therapies manual"[Title/Abstract] OR "therapy manual"[Title/Abstract] OR "Zone Therapy"[Title/Abstract] OR "Zone Therapies"[Title/Abstract] OR "therapy zone"[Title/Abstract] OR "Massage Therapy"[Title/Abstract] OR "Massage Therapies"[Title/Abstract] OR "therapies massage"[Title/Abstract] OR "therapy massage"[Title/Abstract] OR "Shiatsu"[Title/Abstract] OR "Zhi Ya"[Title/Abstract]  #3 #1 OR #2  #4“neoplasm"[Mesh] OR "neoplasm" OR "neoplasm"[Title/Abstract] OR neoplas*[Title/Abstract] OR “tumor*”[Title/Abstract] OR "tumour"[Mesh] OR "tumour" OR tumour[Title/Abstract] OR tumor[Title/Abstract] OR “krebs*”[Title/Abstract] OR “cancer*”[Title/Abstract] OR “malignan*”[Title/Abstract] OR “carcino*”[Title/Abstract] OR “karzino*”[Title/Abstract] OR “karzinom*”[Title/Abstract] OR “sarcom*”[Title/Abstract] OR “leukem*”[Title/Abstract] OR “leukaem*”[Title/Abstract] OR “leucem*”[Title/Abstract] OR “lymphom*”[Title/Abstract] OR “melano*”[Title/Abstract] OR “metastas*”[Title/Abstract] OR “mesothelio*”[Title/Abstract] OR “mesotelio*”[Title/Abstract] OR “carcinomatos*”[Title/Abstract] OR “gliom*”[Title/Abstract] OR “glioblastom*”[Title/Abstract] OR “osteo?sarcom*”[Title/Abstract] OR “blastom*”[Title/Abstract] OR “neuroblastom*”[Title/Abstract] OR oncology[Title/Abstract]  #5“mixed methods” [Title/Abstract] OR "mixed study"[Title/Abstract] OR "mixed research"[Title/Abstract] OR "qualitative research"[Title/Abstract] OR "qualitative study"[Title/Abstract] OR "qualitative methodology"[Title/Abstract] OR "phenomen* research"[Title/Abstract] OR "grounded theory*"[Title/Abstract] OR "ethnograph*"[Title/Abstract] OR "historic research"[Title/Abstract] OR "historic study"[Title/Abstract] OR "action research"[Title/Abstract] OR “focus group” [Title/Abstract] OR “narrative” [Title/Abstract] OR “lived experience*” [Title/Abstract]  #6 #3 AND #4 AND #5 |
| **Embase** | #1"Acupuncture":ti,ab,kw OR "Acupuncture Therapy":ti,ab,kw OR "acupuncture, ear":ti,ab,kw OR "Acupuncture Points":ti,ab,kw OR "Acupuncture Analgesia":ti,ab,kw OR "Electroacupuncture":ti,ab,kw OR "Transcutaneous Electric Nerve Stimulation":ti,ab,kw OR "Acupuncture":ti,ab,kw OR "Electroacupuncture":ti,ab,kw OR "body needling":ti,ab,kw OR "Acupuncture Therapy":ti,ab,kw OR "ear acupuncture":ti,ab,kw OR "auricular needling":ti,ab,kw OR "scalp acupuncture":ti,ab,kw OR "laser acupuncture":ti,ab,kw OR "transcutaneous electrical nerve stimulation":ti,ab,kw OR "moxibustion":ti,ab,kw OR "moxibustion":ti,ab,kw OR "moxa":ti,ab,kw OR "massage":ti,ab,kw OR "acupressure":ti,ab,kw OR "reflexotherapy":ti,ab,kw OR "medicine, ayurvedic":ti,ab,kw OR "aromatherapy":ti,ab,kw OR "Reflexology":ti,ab,kw OR "Rolfing":ti,ab,kw OR "Manual Therapies":ti,ab,kw OR "Manual Therapy":ti,ab,kw OR "therapies manual":ti,ab,kw OR "therapy manual":ti,ab,kw OR "Zone Therapy":ti,ab,kw OR "Zone Therapies":ti,ab,kw OR "therapy zone":ti,ab,kw OR "Massage Therapy":ti,ab,kw OR "Massage Therapies":ti,ab,kw OR "therapies massage":ti,ab,kw OR "therapy massage":ti,ab,kw OR "Shiatsu":ti,ab,kw OR "Zhi Ya":ti,ab,kw  #2“Medicine, Chinese Traditional”:ti,ab,kw OR “Medicine, Traditional”:ti,ab,kw OR “Complementary Therapies”:ti,ab,kw OR “Medicine, Complementary”:ti,ab,kw OR “Alternative Medicine”:ti,ab,kw OR “Drugs, Chinese Herbal”:ti,ab,kw OR “herb*”:ti,ab,kw OR “phytotherapy”:ti,ab,kw OR “complimentary next therap*”:ti,ab,kw OR decoction:ti,ab,kw OR granule*:ti,ab,kw OR pill:ti,ab,kw OR pills:ti,ab,kw OR tablet*:ti,ab,kw OR “traditional CHM” OR “herbal medicine” OR “integrative medicine” OR “Chinese herb” OR “Chinese patent medicine”  #3 #1 OR #2  #4“mixed methods”:ti,ab,kw OR "mixed study":ti,ab,kw OR "mixed research":ti,ab,kw OR "qualitative research":ti,ab,kw OR "qualitative study":ti,ab,kw OR "qualitative methodology":ti,ab,kw OR "phenomen* research":ti,ab,kw OR "grounded theory*":ti,ab,kw OR "ethnograph*":ti,ab,kw OR "historic research":ti,ab,kw OR "historic study":ti,ab,kw OR "action research":ti,ab,kw OR “focus group”:ti,ab,kw OR “narrative”:ti,ab,kw OR “lived experience*”:ti,ab,kw  #5 "neoplas*" OR “tumor*” OR "tumour" OR “krebs*”:ti,ab,kw OR “cancer*”:ti,ab,kw OR “malignan*”:ti,ab,kw OR “carcino*”:ti,ab,kw OR “karzino*”:ti,ab,kw OR “karzinom*”:ti,ab,kw OR “sarcom*”:ti,ab,kw OR “leukem*”:ti,ab,kw OR “leukaem*”:ti,ab,kw OR “leucem*”:ti,ab,kw OR “lymphom*”:ti,ab,kw OR “melano*”:ti,ab,kw OR “metastas*”:ti,ab,kw OR “mesothelio*”:ti,ab,kw OR “mesotelio*”:ti,ab,kw OR “carcinomatos*”:ti,ab,kw OR “gliom*”:ti,ab,kw OR “glioblastom*”:ti,ab,kw OR “osteo?sarcom*”:ti,ab,kw OR “blastom*”:ti,ab,kw OR “neuroblastom*”:ti,ab,kw OR oncology:ti,ab,kw  #6 #3 AND #4 AND #5 |
| **Web of Science** | #1TS=(“Medicine, Chinese Traditional” OR “Medicine, Traditional” OR “Complementary Therapies” OR “Medicine, Complementary” OR “Alternative Medicine” OR “Drugs, Chinese Herbal” OR “herb*” OR “phytotherapy” OR “complimentary next therap*” OR decoction OR granule* OR pill OR pills OR tablet* OR “traditional CHM” OR “herbal medicine” OR “integrative medicine” OR“Chinese herb” OR “Chinese patent medicine”)  #2TS=("Acupuncture" OR "Acupuncture Therapy" OR "acupuncture, ear" OR "Acupuncture Points" OR "Acupuncture Analgesia" OR "Electroacupuncture" OR "Transcutaneous Electric Nerve Stimulation" OR "Acupuncture" OR "Electroacupuncture" OR "body needling" OR "Acupuncture Therapy" OR "ear acupuncture" OR "auricular needling" OR "scalp acupuncture" OR "laser acupuncture" OR "transcutaneous electrical nerve stimulation" OR "moxibustion" OR "moxibustion" OR "moxa" OR "massage" OR "acupressure" OR "reflexotherapy" OR "medicine, ayurvedic" OR "aromatherapy" OR "Reflexology" OR "Rolfing" OR "Manual Therapies" OR "Manual Therapy" OR "therapies manual" OR "therapy manual" OR "Zone Therapy" OR "Zone Therapies" OR "therapy zone" OR "Massage Therapy" OR "Massage Therapies" OR "therapies massage" OR "therapy massage" OR "Shiatsu" OR "Zhi Ya")  #3 #1 OR #2  #4TS=(“neoplasm" OR "neoplasm" OR "neoplasm" OR neoplas* OR “tumor*” OR "tumour" OR "tumour" OR tumour OR tumor OR “krebs*” OR “cancer*” OR “malignan*” OR “carcino*” OR “karzino*” OR “karzinom*” OR “sarcom*” OR “leukem*” OR “leukaem*” OR “leucem*” OR “lymphom*” OR “melano*” OR “metastas*” OR “mesothelio*” OR “mesotelio*” OR “carcinomatos*” OR “gliom*” OR “glioblastom*” OR “osteo?sarcom*” OR “blastom*” OR “neuroblastom*” OR oncology)  #5TS=(“mixed methods” OR "mixed study" OR "mixed research" OR "qualitative research" OR "qualitative study" OR "qualitative methodology" OR "phenomen* research" OR "grounded theory*" OR "ethnograph*" OR "historic research" OR "historic study" OR "action research" OR “focus group” OR “narrative” OR “lived experience*”)  #6 #3 AND #4 AND #5 |
| **CINAHL** | #1"Acupuncture":ti,ab,kw OR "Acupuncture Therapy":ti,ab,kw OR "acupuncture, ear":ti,ab,kw OR "Acupuncture Points":ti,ab,kw OR "Acupuncture Analgesia":ti,ab,kw OR "Electroacupuncture":ti,ab,kw OR "Transcutaneous Electric Nerve Stimulation":ti,ab,kw OR "Acupuncture":ti,ab,kw OR "Electroacupuncture":ti,ab,kw OR "body needling":ti,ab,kw OR "Acupuncture Therapy":ti,ab,kw OR "ear acupuncture":ti,ab,kw OR "auricular needling":ti,ab,kw OR "scalp acupuncture":ti,ab,kw OR "laser acupuncture":ti,ab,kw OR "transcutaneous electrical nerve stimulation":ti,ab,kw OR "moxibustion":ti,ab,kw OR "moxibustion":ti,ab,kw OR "moxa":ti,ab,kw OR "massage":ti,ab,kw OR "acupressure":ti,ab,kw OR "reflexotherapy":ti,ab,kw OR "medicine, ayurvedic":ti,ab,kw OR "aromatherapy":ti,ab,kw OR "Reflexology":ti,ab,kw OR "Rolfing":ti,ab,kw OR "Manual Therapies":ti,ab,kw OR "Manual Therapy":ti,ab,kw OR "therapies manual":ti,ab,kw OR "therapy manual":ti,ab,kw OR "Zone Therapy":ti,ab,kw OR "Zone Therapies":ti,ab,kw OR "therapy zone":ti,ab,kw OR "Massage Therapy":ti,ab,kw OR "Massage Therapies":ti,ab,kw OR "therapies massage":ti,ab,kw OR "therapy massage":ti,ab,kw OR "Shiatsu":ti,ab,kw OR "Zhi Ya":ti,ab,kw  #2“Medicine, Chinese Traditional”:ti,ab,kw OR “Medicine, Traditional”:ti,ab,kw OR “Complementary Therapies”:ti,ab,kw OR “Medicine, Complementary”:ti,ab,kw OR “Alternative Medicine”:ti,ab,kw OR “Drugs, Chinese Herbal”:ti,ab,kw OR “herb*”:ti,ab,kw OR “phytotherapy”:ti,ab,kw OR “complimentary next therap*”:ti,ab,kw OR decoction:ti,ab,kw OR granule*:ti,ab,kw OR pill:ti,ab,kw OR pills:ti,ab,kw OR tablet*:ti,ab,kw OR “traditional CHM” OR “herbal medicine” OR “integrative medicine” OR “Chinese herb” OR “Chinese patent medicine”  #3 #1 OR #2  #4“mixed methods” OR "mixed study" OR "mixed research" OR "qualitative research" OR "qualitative study" OR "qualitative methodology" OR "phenomen* research" OR "grounded theory*" OR "ethnograph*" OR "historic research" OR "historic study" OR "action research" OR “focus group” OR “narrative” OR “lived experience*”  #5 "neoplas*" OR “tumor*” OR "tumour" OR “krebs*”:ti,ab,kw OR “cancer*”:ti,ab,kw OR “malignan*”:ti,ab,kw OR “carcino*”:ti,ab,kw OR “karzino*”:ti,ab,kw OR “karzinom*”:ti,ab,kw OR “sarcom*”:ti,ab,kw OR “leukem*”:ti,ab,kw OR “leukaem*”:ti,ab,kw OR “leucem*”:ti,ab,kw OR “lymphom*”:ti,ab,kw OR “melano*”:ti,ab,kw OR “metastas*”:ti,ab,kw OR “mesothelio*”:ti,ab,kw OR “mesotelio*”:ti,ab,kw OR “carcinomatos*”:ti,ab,kw OR “gliom*”:ti,ab,kw OR “glioblastom*”:ti,ab,kw OR “osteo?sarcom*”:ti,ab,kw OR “blastom*”:ti,ab,kw OR “neuroblastom*”:ti,ab,kw OR oncology:ti,ab,kw  #6 #3 AND #4 AND #5 |
| **知网** | (FT=('中医‘+'中药'+'中医药'+'草药'+'中草药'+'传统医药'+'中成药'+'针灸'+'针刺'+'电针'+'体针'+'耳针+'头针'+'灸'+'按摩'+'推拿'+'点穴'+'穴位按压'+'拔罐'+'刮痧'+'埋线'+'穴位注射') OR SU=('中医‘+'中药'+'中医药'+'草药'+'中草药'+'传统医药'+'中成药'+'针灸'+'针刺'+'电针'+'体针'+'耳针+'头针'+'灸'+'按摩'+'推拿'+'点穴'+'穴位按压'+'拔罐'+'刮痧'+'埋线'+'穴位注射')) AND (SU=('定性研究'+'质性研究'+'访谈'+'民族志'+'人种学'+'扎根理论'+'现象学'+'行动研究法'+'混合研究') OR TKA=('定性研究'+'质性研究'+'访谈'+'民族志'+'人种学'+'扎根理论'+'现象学'+'行动研究法'+'混合研究')) AND (SU=(‘肿瘤’+‘癌’ + ‘瘤’ + ‘恶性’ + ‘白血’ + ‘骨髓’ + ‘淋巴’) OR TKA=(‘肿瘤’+‘癌’ + ‘瘤’ + ‘恶性’ + ‘白血’ + ‘骨髓’ + ‘淋巴’)) |
| **维普** | (U=中医 OR 中药 OR 中医药 OR 草药 OR 中草药 OR 传统医药 OR 中成药 OR 针灸 OR 针刺 OR 电针 OR 体针 OR 耳针 OR 头针 OR 灸 OR 按摩 OR 推拿 OR 点穴 OR 穴位按压 OR 拔罐 OR 刮痧 OR 埋线 OR 穴位注射) AND ((M=肿瘤 OR 癌 OR 瘤 OR 恶性 OR 白血 OR 骨髓 OR 淋巴) OR (R=肿瘤 OR 癌 OR 瘤 OR 恶性 OR 白血 OR 骨髓 OR 淋巴)) AND ((M=定性研究 OR 质性研究 OR 访谈 OR 民族志 OR 人种学 OR 扎根理论 OR 现象学 OR 行动研究法 OR 混合研究) OR (R=定性研究 OR 质性研究 OR 访谈 OR 民族志 OR 人种学 OR 扎根理论 OR 现象学 OR 行动研究法 OR 混合研究)) |
| **万方** | 摘要: ("中医" OR "中药" OR "中医药" OR "草药" OR "中草药" OR "传统医药" OR "中成药" OR "针灸" OR "针刺" OR "电针" OR "体针" OR "耳针" OR "头针" OR "灸" OR "按摩" OR "推拿" OR "点穴" OR "穴位按压" OR "拔罐" OR "刮痧" OR "埋线" OR "穴位注射") and ("定性研究" OR "质性研究" OR "访谈" OR "民族志" OR "人种学" OR "扎根理论" OR "现象学" OR "行动研究法" OR "混合研究") and ("肿瘤" OR "癌" OR "瘤" OR "恶性" OR "白血" OR "骨髓" OR "淋巴") or 全部:("中医" OR "中药" OR "中医药" OR "草药" OR "中草药" OR "传统医药" OR "中成药" OR "针灸" OR "针刺" OR "电针" OR "体针" OR "耳针" OR "头针" OR "灸" OR "按摩" OR "推拿" OR "点穴" OR "穴位按压" OR "拔罐" OR "刮痧" OR "埋线" OR "穴位注射") and ("定性研究" OR "质性研究" OR "访谈" OR "民族志" OR "人种学" OR "扎根理论" OR "现象学" OR "行动研究法" OR "混合研究") and ("肿瘤" OR "癌" OR "瘤" OR "恶性" OR "白血" OR "骨髓" OR "淋巴") |
| **SinoMed** | [( ""中医""[常用字段:智能] OR ""中药""[常用字段:智能] OR ""中医药""[常用字段:智能] OR ""草药""[常用字段:智能] OR ""中草药""[常用字段:智能] OR ""传统医药""[常用字段:智能] OR ""中成药""[常用字段:智能] OR ""针灸""[常用字段:智能] OR ""针刺""[常用字段:智能] OR ""电针""[常用字段:智能] OR ""体针""[常用字段:智能] OR ""耳针""[常用字段:智能] OR ""头针""[常用字段:智能] OR ""灸""[常用字段:智能] OR ""按摩""[常用字段:智能] OR ""推拿""[常用字段:智能] OR ""点穴""[常用字段:智能] OR ""穴位按压""[常用字段:智能] OR ""拔罐""[常用字段:智能] OR ""刮痧""[常用字段:智能] OR ""埋线""[常用字段:智能] OR ""穴位注射""[常用字段:智能]) AND( ""肿瘤""[常用字段:智能] OR ""癌""[常用字段:智能] OR ""瘤""[常用字段:智能] OR ""恶性""[常用字段:智能] OR ""白血""[常用字段:智能] OR ""骨髓""[常用字段:智能] OR ""淋巴""[常用字段:智能]) AND( ""定性研究""[常用字段:智能] OR ""质性研究""[常用字段:智能] OR ""访谈""[常用字段:智能] OR ""民族志""[常用字段:智能] OR ""人种学""[常用字段:智能] OR ""扎根理论""[常用字段:智能] OR ""现象学""[常用字段:智能] OR ""行动研究法""[常用字段:智能] OR ""混合研究""[常用字段:智能])](javascript:toDoRelimitSearch();) |

| Table S3.CASP checklist  Q1: Was there a clear statement of the aims of the research?  Q2: Is a qualitative methodology appropriate?  Q3: Was the research design appropriate to address the aims of the research?  Q4: Was the recruitment strategy appropriate to the aims of the research?  Q5: Was the data collected in a way that addressed the research issue?  Q6: Has the relationship between researcher and participants been adequately considered?  Q7: Have ethical issues been taken into consideration?  Q8: Was the data analysis sufficiently rigorous?  Q9: Is there a clear statement of findings?  Q10: How valuable is the research? |
| --- |

Table S4. Study characteristics

| **Study**  **（First author, Year）** | **Country** | **Aims of the study** | **Sample**  **(N; age range or mean age)** | **Participants** | **Therapeutic Modalities in TCM** | **Health Issues** | **Design/Sampling methods** | **Data collection method** | **Data analysis method** | **Short description of main results** |
| --- | --- | --- | --- | --- | --- | --- | --- | --- | --- | --- |
| Billhult (2007) | Sweden | This study aimed to describe the massage experience for breast cancer patients during chemotherapy treatment. | 10; 50 | Breast cancer patients | Massage | Chemotherapy-Related Issues | Qualitative; Not Specified | Interviews | Giorgi’s phenomenological method | Results revealed five themes: the patients experienced distraction from the frightening experience, a turn from negative to positive, a sense of relaxation, a confirmation of caring, and finally, they just felt good. The findings of this study show that massage offered a retreat from uneasy, unwanted, negative feelings connected with chemotherapy treatment. P.85 |
| Burrows Simpson (2003) | Hong Kong, China | To explore beliefs about diet and traditional Chinese medicine related to the breast cancer experience of Chinese women and their families. | 20; 30-58 | Breast cancer patients | Qi Gong, Herbal Medicine, TCM Dietary Therapy | Unspecified Health Issues | Qualitative; Purposive sampling | Semi-structured interviews | Content analysis | Most participants in this study believed in and encouraged traditional Chinese medicine practices as complementary therapies, primarily for managing symptoms and maintaining health. P.838 |
| Chan (2014) | China | This study aimed to explore the influence of traditional Chinese medicine (TCM) on self-care among Chinese cancer patients. | 30; 28-76 | Cancer patients | Self-care therapies guided by TCM philosophy (e.g., TCM Dietary Therapy, Herbal and Dietary Supplements, Qigong, etc.) | Unspecified Health Issues | Qualitative; Purposive sampling | Semi-structured interviews | Content analysis | Two core categories emerged from their experiences with practicing TCM-supported self-care activities: perceived beneficial effects and concerns about undesirable effects. |
| Chen (2023) | Taiwan, China | This study aims to compare the intention of utilization and experience toward traditional Chinese medicine among early- and late-stage breast cancer patients. | 19; 43-71 | Breast cancer patients | Herbal Medicine, Acupuncture | Unspecified Health Issues | Qualitative; Convenience sampling | Semi-structured focus group interviews | Content analysis | The side effects were the primary intention of utilizing traditional Chinese medicine. Improved side effects and fitness were the main benefits for patients in both phases. |
| De Valois (2016) | UK | To explore how patients with lymphoedema secondary to cancer treatment perceive and experience acu/moxa treatment. | 23; 43-83 | Cancer survivors with lymphoedema | Acupuncture, Moxibustion | Seondary lymphoedema | Qualitative; Not Specified | Focus group interviews | Thematic analysis | Acu/moxa has the potential to benefit some patients with cancer-related upper body lymphedema, improve some other aspects of their symptoms, and facilitate the transition from powerlessness brought on by the consequences of a cancer diagnosis and treatment to feeling empowered to take control of their lives. P.12 |
| Stöckigt (2021) | Germany | The aim of the qualitative part of this mixed methods study was to understand better the subjective perspectives of the patients regarding quality of life during chemotherapy and the perceived effects of acupuncture | 20; 55 | Breast cancer patients | Acupuncture | Chemotherapy-Related Issues | Qualitative; Not Specified | Semi-structured interviews | Content analysis | Most of the women reported that their quality of life during chemotherapy was surprisingly better than they had previously expected. All patients agreed that the acupuncture treatments were relaxing and beneficial. Most patients reported that acupuncture treatments alleviated the symptoms of chemotherapy-induced side effects. In addition, acupuncture helped them helpfully cope with the disease. P.7 |
| Ee (2022) | Australia | This study aims to assess the feasibility and acceptability of acupuncture and other methods as adjuncts to lifestyle interventions (diet and exercise) for weight management in women with breast cancer. | 37; 30-86 | Breast cancer patients | Acupuncture | Weight Control During Breast Cancer Recovery | Qualitative; Convenience and purposive sampling | Focus group; semi-structured interviews | Thematic analysis | Most women find acupuncture too invasive to accept as a weight management method. Acupuncture can be acceptable if there is sufficient education about its benefits. P.12 |
| Eriksen (2018) | USA | To understand cancer survivors’ perceptions regarding the use of acupuncture for the treatment of cancer-related insomnia. | 63; 60 | Patients with cancer-related insomnia | Acupuncture | Cancer-Related Insomnia | Qualitative; Theoretical sampling | Semi-structured interviews | Integrated approach—Codes derived from grounded theory analysis combined with priori codes | Individuals in this study expressed confusion surrounding what symptoms or medical conditions acupuncture could be used to treat, how acupuncture works, and how long the effects of acupuncture last. P.962 |
| Hung (2022) | Hong Kong, China | This study aimed to explore the use and perceptions of BCS using TCM. | 67; Not Specified | Breast cancer patients | Unspecified type | Unspecified Health Issues | Qualitative; Theoretical sampling | Semi-structured interviews | Thematic analysis | Chinese Hong Kong BCS who used TCM reported positive experiences. |
| Kweku Sey (2020) | Australia | This study aimed to quantify massage service provision by Australian cancer services and explore providers’ and survivors’ views about massage services. | 33; 64 | Cancer survivors; Oncology massage service providers | Massage | Unspecified Health Issues | Mixed-method; Convenience sampling and purposive sampling | Focus group; Individual interview | Thematic analysis | Three meta-themes were identified: 1) an under-provision of high-quality massage services; 2) the perceived benefits of massage extend beyond symptomatic relief; and 3) the interrelated barriers to massage service provision and access reflect different values. P.126 |
| Liu (2011) | Taiwan, China | The goal of this study was to understand the experiences of cancer patients undergoing conventional chemotherapy and using TCM at the same time. | 9; 42-63 | Cancer patients | Unspecified type | Unspecified Health Issues | Qualitative; Purposive sampling | Semi-structured interviews | A constant comparative method | The findings identify the biopsychosocial aspects of using a combination of conventional medicine and TCM by Taiwanese patients with a late-stage cancer diagnosis. Most patients use traditional medicine as their primary therapy and TCM as a supplement to reduce the adverse effects of chemotherapy, rebuild energy, and maintain hope. P.501 |
| Liu (2013) | Taiwan, China | This study explored patients' trust in traditional Chinese medicine. | 12; 50.2 | Cancer patients | Unspecified type | Unspecified Health Issues | Qualitative; Purposive sampling | Semi-structured interviews | A constant comparative method | Our first central theme was that participants’ trust in TCM was molded by their cultural background and their own and family members’ experience of using TCM. Our second major theme was that trust in TCM was augmented by the conventional medicine system: this included categories of doubts about TCM’s effectiveness for cancer treatment, cooperation between the medical and TCM departments in the study hospital, and institutional guarantees. P.495 |
| Peter (2014) | UK | To explore the experience of breast cancer patients who either received acupuncture or were allocated to the control group. | 40; >30 | Breast cancer patients | Acupuncture | Unspecified Health Issues | Qualitative; Not Specified | Semi-structured interviews | Thematic analysis | Five common themes were identified, including drivers to take part, the experience of receiving acupuncture, being allocated standard care (control), and reflections on participating in the trial. Recipients of acupuncture reported beneficial effects in managing fatigue and related symptoms. P.291 |
| Mao (2012) | USA | This study seeks to identify attitudes and beliefs about using acupuncture for HFs by BCS. | 25; 57 | Breast cancer patients with hot flashes | Acupuncture | Hot Flashes | Qualitative; Purposive sampling | Semi-structured interviews | A modified grounded theory analysis | BCS expressed varied expected therapeutic benefits, practical concerns, and decision support, emphasizing the “natural appeal” and symptom appraisal as key determinants in using acupuncture for HFs. Incorporating these factors in counseling BCS may promote patient-centered communication, improving hot flash management and quality of life. P.1-2 |
| Murley (2019) | USA | The purposes of this study were to (a) evaluate the effects of TC on self-efficacy, quality of life (QOL), and cancer-related fatigue (CRF) and (b) understand the experience and perceived benefits of patients taking chemotherapy involved in a TC program. | 3; 30-69 | Cancer chemotherapy patients | Tai Chi | Chemotherapy-Related Issues | Mixed-method; Convenience sampling | Semi-structured focus group interviews | Thematic analysis | Tai chi is an effective and feasible exercise for cancer patients receiving chemotherapy. This mind-body exercise appears to provide benefits beyond improved physical functioning. P.1 |
| Oberoi (2022) | Canada | This exploratory study sought to compare patient experiences and acceptability of group versus individual AP in cancer patients. | 11; 57.1 | Patients with Cancer-related pain | Acupuncture | Cancer-related Pain | Qualitative; Purposive sampling | In-depth semi-structured, open-ended interviews | Thematic analysis | Participants across both treatment arms acknowledged improvement in pain, sleep quality, mood, and fatigue. Participants in the group AP arm reported a significant increase in perceived social support, while participants in the individual arm valued privacy and ‑one-on-one interaction with the acupuncturist. P.1 |
| Osypiuk (2020) | USA | This study aimed to explore the perceptions of BCS as they learned the practice of QMBE. | 18; 55 | Breast cancer survivors | Qi Gong | Unspecified Health Issues | Qualitative; Not Specified | Semi-structured interviews | Grounded theory analysis | BCS disclosed a disconnect between mind and body that emerged during treatment. They perceived QMBE as a moving meditation, which enabled them to reconnect mind and body, lessen their pain, and make peace with their bodies. |
| Özdemir (2023) | Turkey | This study aimed to investigate the effect of acupressure on the severity and level of cancer-related fatigue in elderly patients with cancer. | 15; 69.73 | Elderly patients with cancer-related fatigue | Acupressure | Cancer-Related Fatigue | Mixed-method; Not Specified | In-depth semi-structured interviews | Descriptive and content analysis | Acupressure can be recommended as an integrative treatment for cancer-related fatigue because it is effective, easy to use, tolerable, and does not cause serious side effects. |
| Mackereth (2023) | UK | This study aimed to explore the experience of participating in a clinical study of acupuncture for CIPN. | 23; 42-81 | Cancer patients with chemotherapy-induced peripheral neuropathy | Acupuncture | Chemotherapy-Induced Peripheral Neuropathy | Qualitative; Not Specified | Semi-structured interviews | Thematic analysis | Information, motivation, and allocation – participants expressed positive expectations and hoped acupuncture would improve symptoms of CIPN. Acupuncture experiences – participants reported beneficial effects of acupuncture on their CIPN symptoms and quality of life. P.1 |
| Porter (2017) | Australia | This article examines breast cancer survivors’ experiences, perceptions of, and beneﬁts (or not) in using TCM. | 13; 46 | Breast cancer patients | Acupuncture, TCM Dietary Therapy, Herbal Medicine, Qi Gong | Unspecified Health Issues | Qualitative; Not Specified | In-depth semi-structured interviews | Grounded theory analysis | Participants reported perceived outcomes and health benefits from TCM usage ranging from increased coping mechanisms, relieving stress and side-effects of standard treatment, the desire to be proactive in the treatment journey, and having a locus of control. Some cited the importance of “time-out” and the therapeutic relationship with the practitioner. P.1 |
| Price (2013) | UK | This study aimed to explore the needs and concerns of women with early breast cancer during chemotherapy | 14; 54 | Breast cancer patients | Acupuncture | Chemotherapy-Related Issues | Qualitative; Not Specified | In-depth interviews | Grounded theory analysis | The women reported both broad and specific benefits; a highly valued outcome was enabling coping through the alleviation of symptoms and increased well-being. Practitioners dealt with the presented symptom clusters facilitating outcome patterns, including and beyond individual symptom changes. P.1 |
| Price (2014) | UK | This study examined what TA practitioners aim to achieve, their rationale, and how they follow this through in their practice. | 2; Not Specified | Traditional acupuncture practitioners | Acupuncture | Chemotherapy-Related Issues | Qualitative; Theoretical sampling | In-depth interviews, diaries, and treatment logs | Grounded theory analysis | Practitioners aim for long-term goals of increased strength, coping ability, and immediate relief of symptoms. The relapsing and individualized nature of TA is also followed in therapy. At the same time, the practitioners believed that building trust and good relationships with the women would help achieve the goals. P.1 |
| Romero (2020) | USA | This study aimed to identify factors that influence the perception of acupuncture’s therapeutic effect among cancer survivors with insomnia. | 28; 60.1 | Cancer insomnia patients | Acupuncture | Cancer-Related Insomnia | Qualitative; Not Specified | Semi-structured interviews | Grounded theory analysis | Participants perceived the ability to respond to acupuncture as dependent on treatment that effectively: 1) alleviated co-morbidities contributing to insomnia, 2) supported sleep hygiene practices, and 3) provided a durable therapeutic effect. Acupuncture treatment that did not address one of these themes often detracted from positive treatment outcomes and diminished perceived benefit from acupuncture. P.1-2 |
| Schapira (2014) | USA | The objective of this study is to identify key decision-making factors among breast cancer survivors considering entry into an acupuncture clinical trial for the treatment of symptoms. | 25; 38-79 | Breast cancer patients with hot flashes | Acupuncture | Unspecified Health Issues | Qualitative; Purposive sampling | open-ended, semi-structured interviews | Thematic analysis | Six themes emerged reflecting key attributes of the decision to enter a clinical trial: (1) symptom appraisal, (2) practical barriers (e.g., distance and travel), (3) beliefs about the interventions (e.g., fear of needles and dislike of medications), (4) comfort with elements of clinical trial design (e.g., randomization, the nature of the control intervention, and blinding), (5) trust, and (6) altruism. P.1 |
| Walker (2007) | UK | Qualitative research aimed to elicit the opinions of women who received standardized ear acupuncture protocol. | 16; Not Specified | Breast cancer patients with hot flashes and/or night sweats | Ear acupuncture | Hot Flashes | Qualitative; Not Specified | Focus group | Grounded theory analysis | The women, who had all been experiencing chronic multiple flushes and sweats, found the acupuncture helpful and relaxing. Many reported reductions in hot flush frequency and improvements in overall emotional and physical well-being. The group setting for treatment was regarded as supportive and encouraging. P.250 |
| Wang (2014) | China | The study aims to explore cancer survivors’ perspectives on the experience of WM and TCM treatment and rehabilitation. | 68; 42-78 | Cancer survivors | Unspecified type | Unspecified Health Issues | Qualitative; Not Specified | Focus group | Thematic analysis | TCM was primarily used in the recovery phase. The lack of communication between doctors and cancer patients affects treatment adherence and impairs the doctor-patient relationship. The cumulative costs of frequent use of TCM in the long rehabilitation period were also high. Conflicting information about dietary supplements tended to make cancer survivors confused. P.8 |
| Xu (2006) | China | The study aims to explore the perspectives and experiences of Chinese cancer patients and TCM professionals regarding TCM cancer therapies | 28; Not Specified | Cancer patients; TCM professionals | Unspecified type | Unspecified Health Issues | Qualitative; Purposive sampling | Focus group with semi-structured in-depth interviews | Thematic analysis | Patient adoption of TCM for cancer treatment is a culturally rooted self-help strategy that is considered effective, safe, and personalized. Long-term benefits, effectiveness of group therapy, and cost-effectiveness are emphasized. Doctors felt that conducting clinical studies would help to increase the recognition of TCM in the West. P.397 |
| Yu (2012) | China | To describe why cancer patients use Chinese medicine (CM) in terms of their expectations, knowledge, attitudes, and barriers. | 23; Not Specified | Cancer patients | , Massage, Acupuncture, Qigong, Herbal Medicine, TCM Dietary Therapy, Five Elements music therapy | Unspecified Health Issues | Qualitative; Convenience sampling and snowballing | Focus groups and in-depth interviews | Thematic analysis | Patients perceive TCM as having better efficacy, being safe, cheaper, and free of adverse effects, available for long-term use, and that TCM is less expensive. They trusted TCM and used it according to their understanding. P.197 |
| Yu (2022) | China | This study aimed to understand the experience of the investigators and subjects in the clinical trial of Chinese medicine for oncology patients. | 25; 40-79 (P) 9; 23-42 (HCP) | Subjects; Investigators | Unspecified type | Unspecified Health Issues | Mixed-method; Not Specified | Semi-structured in-depth interviews | Thematic analysis | While there were overall commonalities between subjects and researchers regarding the factors affecting adherence and strategies for improvement, there were some differences in perspectives and perceptions. P.153 |
| Hu (2019) | USA | To explore the relationship between acupressure and the experience of well-being among children being treated for cancer who received acupressure. | 13; Not Specified | Caregiver of child with pediatric cancer and acupressure provider | Acupressure | Unspecified Health Issues | Qualitative; Purposive sampling | Semi-structured interviews; Observation; Ethnographic fieldnotes | Thematic analysis | Acupressure can help alleviate the difficulties of the childhood cancer experience by promoting a sense of well-being in both the child and the caregiver. Acupressure brings symptomatic relief and physical relaxation to the child, allowing the caregiver to feel relief and relaxation. P.1 |
| McPherson (2016) | Australia | The research objective was to understand how TCM practitioners view their role in treating breast cancer patients, their methods (usual treatment/ management strategies), and their perceived outcomes. | 12; 30-75 | TCM practitioners | Unspecified type | Unspecified Health Issues | Qualitative; Purposive sampling | Semi-structured interviews | Grounded theory analysis | The main themes reported here are the following: the role of TCM in the biomedical management of breast cancer, TCM strategies for managing breast cancer, and the perceived holistic approach of the TCM practitioner and the importance of a TCM diagnosis in the role of breast cancer care. |
| Li (2023) | China | Understanding the need for Chinese dietary therapy in patients with cancer-caused fatigue. | 15; 27-75 | Patients with cancer-caused fatigue | TCM Dietary Therapy | Cancer-Related Fatigue | Qualitative; Purposive sampling | Semi-structured interviews | Colaizzi's 7-step method | There is a diversity of patients' needs for the therapeutic role of Chinese dietary medicine, and there is a lack of guidance on scientific dietary knowledge for fatigued patients in the clinic and a lack of correct sources of information on dietary therapy for patients. P.22 |
| Qin (2020) | China | To find out the knowledge of postoperative colorectal cancer patients receiving integrated Chinese medicine treatment about the causes of their disease, their knowledge of Chinese medicine, and the lifestyle changes they made during treatment. | 13; 43-78 | Postoperative colorectal cancer patients | Unspecified type | Unspecified Health Issues | Mixed-method; Purposive sampling | Semi-structured in-depth interviews | Framing Analysis | TCM helps to strengthen immunity and improve patients' mentality, which contributes to the prognosis of the disease and enables patients to return to their families, society, and life better. P.83 |
| Song (2014) | China | Understanding lung cancer patients' needs and real experiences in Chinese medicine care. | 9; 47-68 | Lung cancer patients | Unspecified type | Unspecified Health Issues | Qualitative; Purposive sampling | Semi-structured in-depth interviews | Colaizzi's 7-step method | Most patients lacked knowledge of disease-related TCM care and could not perform some of the TCM technical operations on their own. P.2853 |
| Wang (2010) | China | To gain an in-depth understanding of the objectives of postoperative colorectal cancer patients seeking integrated Chinese medicine treatment, their knowledge, attitudes, and expectations of Chinese medicine treatment, as well as their experiences, beliefs, and possible problems and barriers to Chinese medicine treatment. | 18; 40-70 | Postoperative colorectal cancer patients | Herbal Medicine | Unspecified Health Issues | Mixed-method; Purposive sampling | In-depth interviews | Thematic analysis | Colorectal cancer patients, due to the influence of traditional culture or family background, or due to the recommendation of others, will choose Chinese medicine treatment, the main purpose of which is to alleviate the adverse effects of Western medical treatment and improve the quality of life. P.36 |
| Xie (2017) | China | Exploring the true feelings of children with acute leukemia about acupoint therapy during chemotherapy treatment. | 14; 9.01 | Children with acute leukemia undergoing chemotherapy | Acupressure | Chemotherapy-Related Issues | Qualitative; Purposive sampling | Semi-structured in-depth interviews | Colaizzi's 7-step method | After the acupressure and auricular seeding TCM treatments, the children's physiological comfort level increased, their negative emotions decreased, and they felt cared for by the nursing staff during the treatment process, thus increasing their confidence in the treatment. P.2357 |
| Yin (2017) | China | Exploring the authenticity of malignant tumor chemotherapy patients' experience of Chinese medicine nursing techniques. | 10; 50-77 | Malignant tumor chemotherapy patients | Unspecified type | Chemotherapy-Related Issues | Qualitative; Purposive sampling | Semi-structured in-depth interviews | Colaizzi's 7-step method | Chemotherapy patients have four main problems in accepting TCM nursing techniques: eagerness to see the effects of use, passive acceptance, lack of continuity in observation and evaluation, and financial pressure. P.65 |
| Zhang (2020) | China | Exploring the factors affecting compliance with auricular acupressure in chemotherapy patients with lung cancer. | 11; 39-67 | Lung cancer chemotherapy patients | Unspecified type | Unspecified Health Issues | Mixed-method; Maximum variation sampling | Semi-structured in-depth interviews | Colaizzi's 7-step method | Auricular pressure therapy improves sleep quality, anxiety levels, and quality of life in lung cancer chemotherapy patients. P.37 |
| Zhang (2013) | China | Deep understanding of tumor patients' attitudes, experiences, beliefs about treatment, etc. | 8; 38-75 | Colorectal cancer patients | Ear acupuncture | Chemotherapy-Related Issues | Mixed-method; Purposive sampling | Semi-structured interviews | Thematic analysis | The efficacy of Chinese medicine's comprehensive treatment for advanced colorectal cancer is mainly reflected in improving physical symptoms and quality of life. P.35 |

Table S5. Qualitative synthesis: facilitators and barriers affecting TCM practice

| **Key theme** | **Domain in the Theoretical Domains Framework** | **Facilitators (F) and Barriers (B)** | **Illustrative excerpts supporting the themes** |
| --- | --- | --- | --- |
| **Capability** | **Pt** | | |
|  | **Memory, attention, and decision processes (n=12)** | Integrated Information Processing and Autonomous Decision-making (F)^42,49,64^ | I may ask my husband or three children, but the final decision is mine. SubjectE:65yrs, Caucasian, College Education |
|  |  | Decision-making exploration of alternatives (F)^30–34,50,51,60,65^ | I wasn’t sleeping, and I had night sweats, hot flashes, and the whole work. I tried everything they suggested, like a cold bath. You name it, I’ve taken it, and nothing was helping. At this stage, I was quite happy to take on anything. (Participant 7) |
|  | **Knowledge (n=35)** | Lack of knowledge about TCM (B)^30,32,33,35–38,42,49–51,54,60,62^ | I don’t know how it would work, Subject S: 56yrs, African American, College Education |
|  |  | TCM benefits and trust construct (F)^31–40,42,44–49,52–54,57,62–65,67,68^ | When I had my radiation therapy, I unfortunately got very, very bad burns and the hospital offered me acupuncture for pain management and it worked. I thought it was great. (ID23, age 56 years, FG2) |
|  |  | Uncertainty about TCM efficacy and course of treatment (B)^30,32–34,37,38,40,42,49–51,53,55,66–68^ | Quote 2b: “I haven’t really noticed any kinds of changes. An average pain level I guess would be about a six”. P116 (Individual) |
|  |  | Asymmetric Information in Healthcare (B)^35,48,51,55^ | I didn’t get a chance to discuss this with [Acupuncturist], and I wanted to know on a maintenance program how often it should be. - Female, 66, Breast cancer |
|  | **Skills (n=4)** | Skills for self-management (F)^46,52,53^ | "I applied it easily. I did not experience any difficulty” (Mr. M. T.)." |
|  |  | Healthcare Provider Shortcomings in Oncology Care Specialization (B)^56^ | I couldn't find a decent massage therapist who could work with this as I have no secondaries or pain as such, but who understood cancer, scars, and lack of skin sensation. |
|  | **Behavioral regulation (n=5)** | Promoting Lifestyle Adjustment (F)^35,41,50,64^ | They felt that acupuncture gave them a holistic view of growth and development by focusing on mind, body, and spirit. ……“N”—“I am not willing to put up with a lot of the stresses I used to put up with. I make sure I have time to rest and time for me. Before I would put my health last, but I don’t put work ahead of me anymore”. |
|  |  | Regular Practice of TCM Interventions (F)^35,38,46^ | I cooked soup once every two days and added corn, pear, Chinese pearl barley, or fish maws into the soup. Radiotherapy made me dehydrated because the treatment is of the nature of dryness and heat. The pain from a sore throat hinders me from drinking water. The effects of these kinds of soups are moistening and yin-enriching (滋阴). (RF2) |
|  | HCP | | |
|  | **Knowledge (n=8)** | Perceived benefits of TCM (F) ^32,54,56–60,69^ | Yes, some of our clinicians are aware that massage can assist in reducing pain. |
|  |  | Lack of knowledge about the benefits and applications of TCM (B)^32,56^ | Certain medical staff in the broader organization are not supportive [of massage]. Lack of awareness about the benefits for people to advocate for such a service. |
|  |  | Lack of evidence-based information source (B)^32,56^ | P Have seen patient benefits but am unaware of evidence. |
|  | **Behavioral regulation (n=1)** | Lack of interdisciplinary communication (B)^32^ | I have sent letters stating my treatment, but I don’t really get a response. |
| **Motivation** | **Pt** | | |
|  | **Social/professional role and identity (n=10)** | TCM Choices under Cultural Beliefs (F)^31,48,51,57,64,66^ | TCM is definitely useful. It must be good, otherwise Chinese people would not have kept using it for 5000 years. |
|  |  | Physician authority and patient trust (F)^34,63,67^ | To me, the most important is to cooperate with doctors, without too many opinions. Doctors are here to cure you, so you have to cooperate with them. |
|  | **Belief about capabilities (n=6)** | Desire for a sense of control (F)^41,44,56^ | When offered the opportunity to take part in a trial of oncology massage for prostate patients I agreed. Although not severe at the time I was finding the effects of fatigue very intrusive in my life and felt a little disoriented and not quite in control. |
|  |  | Confidence in the ability to make self-medical choices (F)^33,42,57^ | "I already trust TCM very much, I don't need WM to give narration, so I don't need to ask them. (E2)” |
|  | **Belief about consequences (n=20)** | Positive expectations of TCM's supportive therapeutic effects (F)^33–35,39–42,44,50,54,55,57^ | [After chemotherapy], I needed TCM for follow-up re-building I. To rebuild body energy, I believe more in oriental medicine, especially TCM. |
|  |  | Negative expectations of TCM efficacy and safety(B)^32,35,37,38,42,44,51,53,55,66^ | From my understanding, you just have a bunch of needles and you have certain places, which have proven to work on different areas, whether it’s sleep or whatever. So I don’t think that there’s a lot of—I don’t think that there would be a lot of long-term effects with the acupuncture. —Female Participant, Age 45 |
|  |  | Concerns about product transparency (B)^35,48^ | There are many over-the-counter health products which claim to be developed based on TCM formulae, but we cannot be sure of the amount of the ingredient, also whether the manufacturer has mixed other ingredients into these products. (CF6) |
|  |  | Anticipated adverse effects of conventional treatment (F)^31,39,42,57^ | We prefer to seeing CM doctors, for it does not hurt, . . . and WM has a strong adverse effect.” (FGH, 43, male, carcinoma of esophagus) |
|  |  | Cross-cultural Imitation Belief (F)^49^ | I don’t know how it would work, but it’s been working for the Chinese, I guess it would work for me. SubjectS:56yrs, African American, College Education |
|  | **Emotions (n=19)** | Disease-related negative emotions (F)^30,40,42,44,47,50,56,60,64^ | As one participant said, ‘‘I had this giant ball of stuck anxiousness. The pain causes anxiety, I can’t calm my brain. My anxiety is paralyzing. |
|  |  | Negative emotions about conventional treatment (F)^31,33,39,44,47,49,62,63^ | I hate taking pills. I have enough of ‘em to take as it is. SubjectK:69yrs, African American, College Education |
|  |  | TCM Treatment-Related Negative Experience (B)^31–33,35,36,40,49,50,62,64^ | I feel like my body’s been through too much for me to stick needles into it again. [laughs] I think my body is just a bit too sensitive at this point (ID47, age 39 years, FG5) |
|  | **Goals (n=15)** | Comprehensive treatment pursuit (F)^31,33,39,41,42,44,51,56,57,62–65^ | TCM is a very good supplement to consolidate and potentiate the treatment effect and reduce the adverse effects of chemotherapy. But in most situations, I don’t think TCM alone can cure cancer. (Meeting 2, patient D) |
|  |  | Security Seeking in the Context of Limited Healthcare Resource (F)^66^ | There was no available ward in the conventional medical department. Besides, it’s too far from my home. I came to the TCM department just to get a bed. |
|  | **Intentions (n=6)** | Recommendation behavior is driven by efficacy satisfaction (F)^40,44,46,64^ | One participant mentioned that she had been “going to [conventional] treatment after treatment for just the main things” and would like to go for AP for relief from symptoms occurring as a side effect of cancer treatment. Having already recommended AP treatment to her father, she said she would even recommend it to others. |
|  |  | Positive TCM experiences drive alternative treatment choices (F)^34,64^ | Of course, TCM is better. It’s totally different from conventional medicine. So after receiving TCM therapy, I left all antiemetics alone. |
|  | **Reinforcement (n=7)** | Positive experiential reinforcement (F)^40,46,54,63,64^ | Quote 7a: “Yeah, that’s I have no idea what it costs. So, but you know my husband would say go for it, it doesn’t matter what it costs just, go for it because it helps so much. So, yeah.” P107 (Group) |
|  |  | Enhanced self-efficacy promotes timely health care behavior (F)^33^ | Normally I ‘put off ’ going to the doctor until things are really bad. This time I had the confidence to request an urgent appointment and get prompt treatment. I feel this is due to the acupuncture/moxibustion treatment I received. |
|  | **HCP** | | |
|  | **Emotions (n=1)** | TCM Treatment-Related Negative Emotions (B)^60^ | Y6: If the taste of this medicine is very bitter, some patients will not want to take it. |
| **Opportunity** | **Pt** | | |
|  | **Social influences (n= 26)** | Significant others' recommendations and endorsement (F)^30,32,35,41,42,44,49,51,53,57,63–66^ | "As for Chinese medicine, an example comes from my mom. She was diagnosed with gastric cancer at the age of 70. From then on, she began to take Chinese medicine. She has taken it for years and now she is 100 years old. [n4fg5, male, colorectal cancer]" |
|  |  | The positive impact of the media (F) ^32,49,64,65^ | Participants had heard good reports of acupuncture [5FBC; 50FGIC; 15MBA; 59FBA] and hoped acupuncture would be beneficial for CIPN. |
|  |  | Harmonized therapeutic relationship (F)^30,32–34,39–41,43–45,50,52,63,65,67,68^ | [The TCM practitioner] is kind and familiar when communicating with patients, like a friend you’ve had for many years. I feel happy to see him. |
|  |  | Positive Social Impact of Patient Support (F)^40,43,47,50,57,68^ | I thought it was beneficial actually because cancer tends to cut you off when you see people in the clinics, and on the wards, but here it was just so nice to know that these are people that are leaving little drips everywhere. You weren’t alone and you could sit and laugh and that did help as well Anything that breaks down the barriers that either you or the disease puts up, all the better. (Participant 2) |
|  |  | Negative or conservative attitudes of physicians (B)^32,36,41,42,44,57,63^ | others have said the oncologist said ‘No, don’t have any needles into your skin at all while you’re having chemotherapy’ (ID14, age 70 years, FG2) |
|  | **Environmental context and resources (n=21)** | The facilitating role of a comfortable environment (F)^40,45^ | Nice environment, and having a kind and pleasant person to talk to (the acupuncturist) while receiving the treatment was a big factor in uplifting their spirits. |
|  |  | Authority of the consultation environment (F)^66^ | “This medical center has its own reputation, and can guarantee patients’ rights ...but in some private clinics, you cannot protect your own rights if there are some medical-legal problems.” |
|  |  | Economic accessibility and policy support (F)^31,34,36,41,56,57^ | “K”—“Absolutely no limitations. Benefits are paid from my private health fund, no problem getting to appointments” |
|  |  | Deceptive Advertising (B)^30^ | The dietary supplements are all described as they can heal any disease, including cancer. And we don’t believe that. Even if we believe it, we can’t afford to buy every product which costs thousands of yuan. [n10fg8, female, lymphoma] |
|  |  | High Treatment Costs (B)^30,31,40,44,48,57,62^ | “M”-The fee was such a burden... I initially went to the TCM practitioner my family members recommended, which was very expensive, and I couldn't afford it. The consultation fee was HKD500, with each dose costing HKD150-170. |
|  | **HCP** | | |
|  | **Social influences (n=2)** | Negative attitudes of oncologists toward the use of TCM (B)^32,57^ | Many [conventional] doctors don’t believe that TCM treatments have an effect on cancer, and thus would not suggest it to patients. Patients themselves make their own decision to use either TCM or conventional therapy, or both. (Professional meeting, Dr Y) |
|  | **Environmental context and resources (n=3)** | Excessive cost of treatment (B)^57^ | Cost - patient chooses not to pursue [massage] treatment options overall. |
|  |  | Lack of specialists (B)^56^ | We do not have a trained staff member nor have funding to access a trained oncology massage therapist. |
|  |  | Limits on the healthcare budget (B)^56^ | Also fundraising pays for the [TCIM] programs but this is a difficult funding stream for sustainability [of a service]. |
|  |  | Lack of communication with biomedical healthcare providers (B)^32^ | Participant (E3) stated, In the US and China I have had a dialogue with oncologists in regard to research.  When probed about the nature of the participant’s (E3) relationship with oncologists in Australia: I have sent letters stating my treatment, but I don’t really get a response. |

Table S6. Subgroup analysis

| **TCM Therapy** | **Unique Facilitators Factors** | **Unique Barriers Factors** |
| --- | --- | --- |
| **Acupuncture** | / | Pt- High Treatment Costs (Time-Intensive and Financially Burdensome)^40,62^ |
|  |  | Pt- TCM Treatment-Related Negative Experience (Needle phobia^32,33,36^, body exposure^39,59^) |
|  |  | Pt- Negative expectations of TCM efficacy and safety (Stimulate tumor spread or progression^57^, Uncertain long-term efficacy^37,38^) |
| **Acupressure** | Pt- Regular Practice of TCM Interventions (Easy to learn and practice regularly)^46^ | Pt- TCM Treatment-Related Negative Experience (Pain and discomfort sensation^46,52^) |
| **Massage** | / | HCP - High Treatment Costs (Financially Burdensome)^56^ |
| **Herbal Medicine** | HCP - Perceived benefits of TCM (Herbs with anticancer properties)^69^ | Pt- High Treatment Costs (Time-Consuming Preparation and Long-Term Use)^30,31,44^ |
|  | Pt- Positive TCM experiences drive alternative treatment choices^34,64^ | Pt- TCM Treatment-Related Negative Experience (Unpleasant taste)^64^ |
|  | / | HCP/Pt- Negative expectations of TCM efficacy and safety (herb/drug interactions)^48,69^ |
| **Nonprescribed Functional Herbal Products** | Pt- Regular Practice of TCM Interventions (Diverse forms and easy accessibility)^35^ | Pt- Deceptive Advertising^30^ |
|  |  | Pt- TCM Treatment-Related Negative Experience (Unpleasant taste)^35^ |
|  |  | Pt- Concerns about product transparency (Production and ingredient transparency)^35,48^ |
|  |  | Pt- Asymmetric Information in Healthcare (Lack of self-care implementation guidance)^35,48,51^ |
|  |  | Pt- High Treatment Costs (Expensive standardized herbal products)^44,57^ |
| **Mind-Body Therapies (e.g., Dynamic types: Qigong, Tai Chi, etc.**  **Static types: Five Elements music therapy, etc.)** | Pt- Perceived Benefits of TCM (Enhancing Mind-Body Integration)^41,47^ | Pt- TCM Treatment-Related Negative Experience (Noise disturbance)^31^ |
|  | Pt- Regular Practice of TCM Interventions (Enhances motor function with moderate intensity)^35^ |  |
| **Herbal footbaths** | / | / |
| **Moxibustion** | / | / |
